# Bienzyme-powered nanorobots with ultrasensitive chemotaxis for precision cancer therapy

**DOI:** 10.1093/nsr/nwaf580

**Published:** 2025-12-18

**Authors:** Zili Yang, Ziye Pei, Zhixue Gao, Ming Luo, Xingchi Liu, Jie Guo, Huanyu Jiang, Mengting Lv, Zili Yu, Suling Zhao, Jianguo Guan

**Affiliations:** State Key Laboratory of Advanced Technology for Materials Synthesis and Processing, International School of Materials Science and Engineering, Wuhan University of Technology, Wuhan 430070, China; State Key Laboratory of Advanced Technology for Materials Synthesis and Processing, International School of Materials Science and Engineering, Wuhan University of Technology, Wuhan 430070, China; State Key Laboratory of Advanced Technology for Materials Synthesis and Processing, International School of Materials Science and Engineering, Wuhan University of Technology, Wuhan 430070, China; State Key Laboratory of Advanced Technology for Materials Synthesis and Processing, International School of Materials Science and Engineering, Wuhan University of Technology, Wuhan 430070, China; Wuhan CyneMed Technology Co., Ltd, Wuhan 430207, China; State Key Laboratory of Oral and Maxillofacial Reconstruction and Regeneration, Key Laboratory of Oral Biomedicine Ministry of Education, Hubei Key Laboratory of Stomatology, School and Hospital of Stomatology, Wuhan University, Wuhan 430079, China; Department of Oral and Maxillofacial Surgery, School and Hospital of Stomatology, Wuhan University, Wuhan 430079, China; State Key Laboratory of Advanced Technology for Materials Synthesis and Processing, International School of Materials Science and Engineering, Wuhan University of Technology, Wuhan 430070, China; State Key Laboratory of Advanced Technology for Materials Synthesis and Processing, International School of Materials Science and Engineering, Wuhan University of Technology, Wuhan 430070, China; State Key Laboratory of Advanced Technology for Materials Synthesis and Processing, International School of Materials Science and Engineering, Wuhan University of Technology, Wuhan 430070, China; State Key Laboratory of Oral and Maxillofacial Reconstruction and Regeneration, Key Laboratory of Oral Biomedicine Ministry of Education, Hubei Key Laboratory of Stomatology, School and Hospital of Stomatology, Wuhan University, Wuhan 430079, China; Department of Oral and Maxillofacial Surgery, School and Hospital of Stomatology, Wuhan University, Wuhan 430079, China; Center for Materials Research and Analysis, Wuhan University of Technology, Wuhan 430070, China; State Key Laboratory of Advanced Technology for Materials Synthesis and Processing, International School of Materials Science and Engineering, Wuhan University of Technology, Wuhan 430070, China; Wuhan CyneMed Technology Co., Ltd, Wuhan 430207, China

**Keywords:** nanorobots, chemotaxis, active targeting, deep penetration, tumor

## Abstract

Low tumor-targeting delivery efficiency (*Ɛ*) and poor tumor penetration remain critical issues in the clinical translation of nanoparticle-based drug delivery systems. Here we report that bienzyme-powered Janus nanorobots with catalase and urease covering the same hemispheres in sequence, demonstrate chemical propulsion far exceeding translational Brownian forces and torques comparable to rotational Brownian torques by leveraging endogenous urea and H₂O₂ gradient in the tumor microenvironment, showcasing ultrasensitive chemotaxis toward biomarkers over-expressed by tumor tissues centimeters away and augmented *Ɛ*. After intravenous injection into a tumor-bearing mouse model, the nanorobots demonstrate significant enhancement in *Ɛ*, penetration depth, and cell internalization, surpassing those of passive counterparts by 209, >10, and 1970 times, respectively. When loaded with antitumor drugs, they boost tumor suppression efficacy by ∼49 times compared with passive counterparts. This work offers a new strategy for next-generation drug delivery, promising a paradigm shift for self-propelled nanorobots in precision medicine.

## Teaser

By decoupling propulsion and orientation, bienzyme-powered nanorobots in tumor microenvironments demonstrate ultrasensitive chemotaxis, achieving markedly improved tumor targeting and deep penetration. After loading with anti-tumor drugs, they show excellent therapeutic efficacy.

## INTRODUCTION

Nanoparticle-based drug delivery systems show promise for cancer therapy by maximizing treatment efficacy while minimizing off-target toxicity [1–3]. Despite decades of endeavor, current systems, such as liposomes, polymeric micelles, mesoporous silica (mSiO_2_), gold (Au) nanoparticles (AuNPs), and dendrimers, still suffer from systemic sequestration and poor tumor penetration [4–10]. Most of them are sequestered by normal tissues and cells, and only ∼0.7% (median) of injected dose (ID) is delivered to tumors [9,10]. Due to the poor penetration capability, <0.0014% of injected NPs are delivered to tumor cells, and <2.0% of tumor cells interact with NPs [11]. This low tumor-targeting delivery efficiency (*Ɛ*) and poor tumor penetration represent critical issues in their clinical translation. To improve *Ɛ*, intensive attempts have been carried out, including improving the physicochemical properties of nanocarriers, or modifying them with an active targeting moiety such as a certain ligand or antibody, increasing the ID of nanocarriers, or reducing hepatic uptake by using biological techniques such as ablating or saturating Kupffer cells [12–18]. Nevertheless, they have only shown a limited increase in *Ɛ*. This underscores the persistent challenges in the field, necessitating further research and development to enhance *Ɛ*.

Moreover, a recent avenue of targeted delivery nanocarrier development has delved into the nuanced microenvironments prevalent in tumors, including features such as tumor hypoxia, low pH, elevated hydrogen peroxide (H_2_O_2_) concentrations, and other distinctive biomarkers [19–21]. These advancements predominantly revolve around the availability of artificial chemically-propelled nanorobots that use the chemicals characteristically overexpressed by tumor cells as fuel and show chemotaxis toward the chemical concentration gradients [22–25]. These nanorobots represent a departure from physical targeted drug delivery nanocarriers that necessitate human intervention to manipulate external physical fields, including light, heat, magnetism, electricity, and ultrasonic forces [26–32]. They can autonomously navigate complex and dynamically evolving biological environments in response to chemical concentration gradients, enabling them to seek out and accumulate at disease sites [33,34]. Thus, they exhibit an inherent level of autonomy and intelligence. Diverging from active targeting delivery systems grounded in ligand-receptor specific interactions, chemically propelled nanorobots operate within a targeting range delimited by the presence of a chemical concentration gradient, typically extending up to several centimeters [16,35]. They manifest remarkable *in vitro* chemotactic mobility toward areas characterized by elevated concentrations of chemoattractants such as reactive oxygen species (ROS) [17], inducible nitric oxide synthase (iNOS), glutathione (GSH), and variations in pH [16,25,36–39], as the asymmetric chemical reactions of the nanorobots with surrounding chemical concentration gradients generate both self-propulsion forces (*F_p_*) and self-orientation rotational torque (*M_p_*), respectively [40]. However, the chemoattractant concentrations at disease sites *in vivo* are generally too low, exemplified by the H_2_O_2_ concentration of ∼50–100 μM in tumor microenvironments [41], to effectively propel nanorobots within physiological or pathological environments. In fact, many previously reported chemical nanorobots show only limited enhancement in *in vivo* tumor-targeting efficiency when compared with their passive counterparts (Table S1) and inadequate penetration ability within solid tumors.

**Figure 1.**
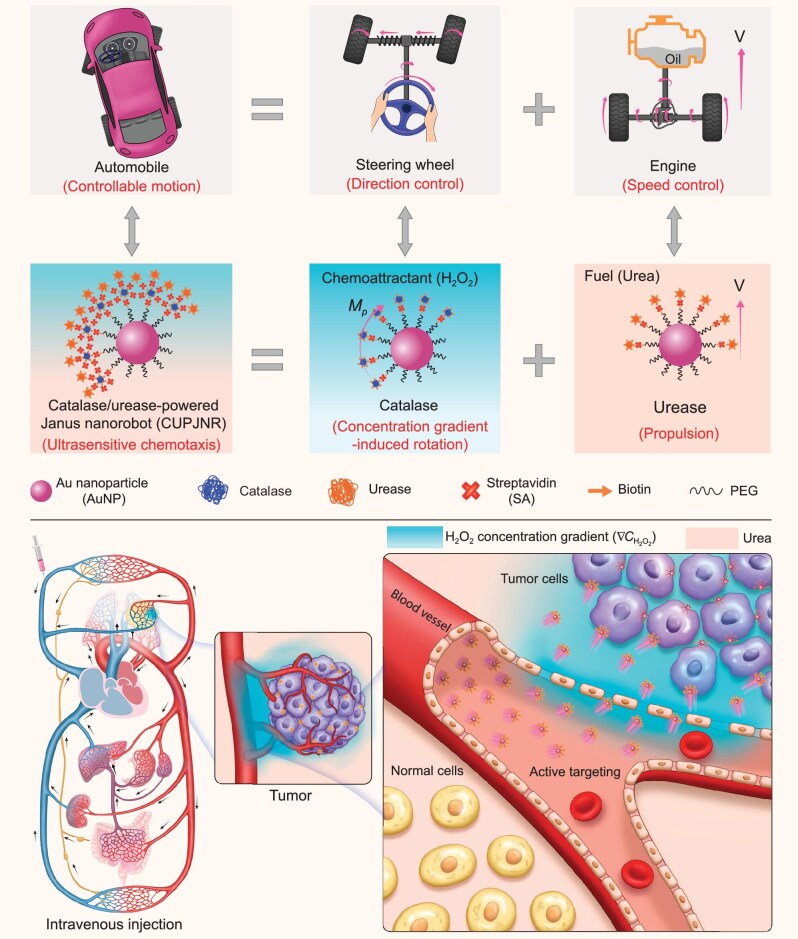
Schematic illustration of the strategy for addressing the issues of low tumor-targeting delivery efficiency and poor penetration capability by switching to catalase/urease-powered Janus nanorobots (CUPJNRs). With a steering wheel to navigate and an engine to power, an automobile can quickly move to a desired destination. In analogy, CUPJNRs can efficiently target tumors, where catalase works for sensing the H_2_O_2_ concentration gradient overexpressed by tumor cells for direction control, and urease serves as the engine using endogenous urea in the bloodstream for propulsive forces. With the synergistic effect of these two enzymatic reaction systems, propulsion and orientation are decoupled in CUPJNRs for respectively overcoming ${\mathop F\limits^{\rightarrow} }_B( t )$ and ${\mathop M\limits^{\rightarrow} }_B( t )$, offering significantly enhanced chemotactic sensitivity toward tumor-specific biomarkers H_2_O_2_. After intravenous injection into a tumor-bearing mouse, the nanorobots can autonomously search for tumors and penetrate deeply there, thus resulting in high tumor-targeting delivery efficiency, deep penetration, and significant cell internalization.

Inspired by automobiles combining an engine and a steering wheel, here we report Janus nanorobots integrating two kinds of enzymes with distinct functionalities to control propulsion and orientation, respectively (Fig. 1). Specifically, we integrated catalase and urease onto the same hemispheres of Au nanoparticles (AuNPs) to develop Janus nanorobots, named as CUPJNRs. Unlike most previously reported dual-enzyme systems that primarily rely on cascade reactions to accelerate catalytic reaction rates [42,43], our urease/catalase system performs two distinct and complementary functions. Urease works for much stronger propulsion forces than translational Brownian forces (${\mathop F\limits^{\rightarrow} }_B( t )$) by harnessing endogenous urea in bloodstream and tumor microenvironments, while catalase for orientation torques are comparable to rotational Brownian torques (${\mathop M\limits^{\rightarrow} }_B( t )$) which are enabled by sensing the H_2_O_2_ concentration gradient typically existing in tumor microenvironments. With this synergistic effect of such two enzymatic reaction systems, the nanorobots in physiological settings exhibit ultrasensitive chemotaxis toward tumor cells, almost independent of the liquid flow. After intravenous administration into a tumor-bearing mouse model, CUPJNRs were able to overcome the critical biological barriers within ‘CAPIR’ (Circulation, Accumulation, Penetration, Internalization, and Release [44]) cascade stages, achieving high *Ɛ*, deep penetration, and significant cell internalization. As delivery carriers, they significantly increase the bioavailability of drugs, thus markedly improving their antitumor efficacy. This study redefines the limits of active targeting, offering a blueprint for intelligent nanorobots in precision oncology.

## RESULTS AND DISCUSSION

### Characterizations and *in vitro* tumor targeting accumulation of CUPJNRs

**Figure 2.**
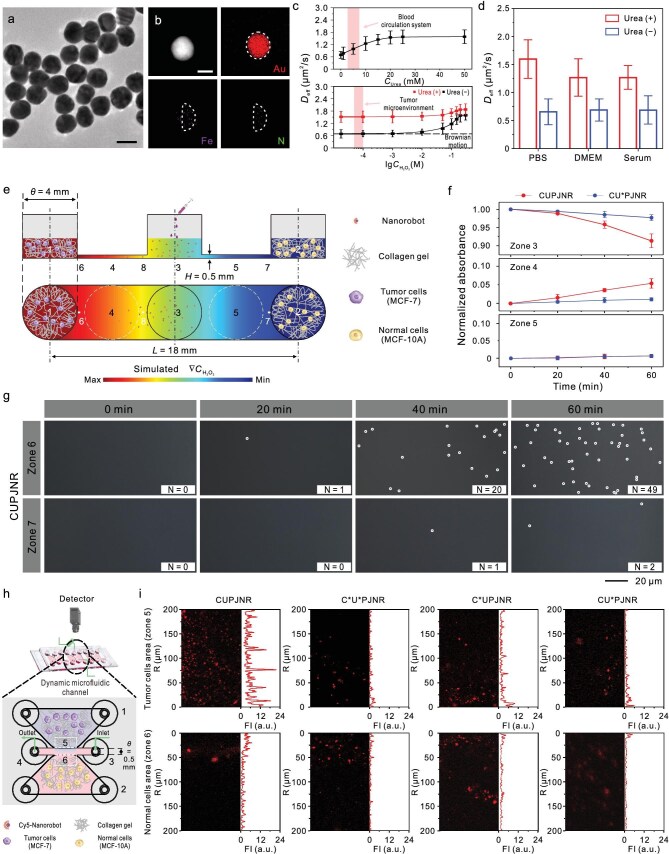
Characterization and targeting accumulation at tumor cells of CUPJNRs. (a and b) Structural characterization. (a) TEM image of CUPJNRs. (b) HAADF-STEM image and EDX mapping analysis of an individual CUPJNR. Scale bar: 50 nm. (c and d) Motion behavior characterization. The diffusion coefficients (*D*_eff_) of CUPJNRs in (c) urea aqueous solution, H_2_O_2_ aqueous solution, and the mixed solution of urea and H_2_O_2_ [*n* = 10; mean ± standard deviation (SD)]. Urea (+) and urea (−) represent the presence and absence of 20 mM urea in solutions. (d) The *D*_eff_ of the CUPJNRs in various biological media (including PBS buffer, DMEM, and serum) [*n* = 10; mean ± SD]. (e–g) Targeting behavior toward tumor cells of CUPJNRs in a microfluidic chip-based three-dimensional (3D) cell model. (e) Schematic illustration of the static microfluidic chip structure, with the background color indicating the simulated H_2_O_2_ concentration. (f) The normalized absorbance at 530 nm of CUPJNRs and CU*PJNRs at zones 3, 4, and 5 indicated in (e) at different time points [*n* = 3; mean ± SD]. (g) Dark-field images of zones 6 and 7 in (e) at different time points. The nanorobots were labelled with white circles for ease of observation. (h and i) Targeting behavior toward tumor cells of CUPJNRs in a dynamic fluid environment. (h) Schematic illustration of the dynamic microfluidic chip structure. (i) The representative confocal fluorescence images and corresponding average fluorescence intensity (FI) at zones 5 and 6 in (h) using different nanorobots modified with Cy5 (red) [*n* = 4]. The enzyme marked with an asterisk is inactive.

The CUPJNRs consisted of AuNPs with catalase and urease asymmetrically attached to the half surfaces in sequence. They were fabricated in two steps, as illustrated in Fig. S1. The first step involved the preparation of biotin-modified Janus AuNPs using a stepwise coupling method according to our previous work (Figs S2−S4) [45]. In the second step, we assembled different biotinylated enzymes onto the surface of biotin-modified Janus AuNPs by employing streptavidin (SA) as a cross-linker [46]. The resulting CUPJNRs preserved the morphology of the original AuNPs and had good dispersibility, as shown in Fig. 2a and Fig. S5. The asymmetrically distributed signal of N element on the CUPJNR suggests that the enzymes were only immobilized on one side of AuNP, while the signal of an Fe element indicates the presence of catalases (Fig. 2b). The number of catalases and ureases on CUPJNR was ∼9 and 5, respectively (Fig. S6). Dynamic light scattering (DLS) and atomic force microscopy (AFM) results suggest that the catalases and ureases were assembled onto the surface of biotin-modified Janus AuNPs via a layer-by-layer mode (Figs S7 and S8). The Janus attachment of the enzymes was further confirmed by high-resolution TEM (Fig. S9). Compared with PEG-modified AuNPs, the polymers were asymmetrically distributed on the surface of CUPJNR, suggesting that the catalases and ureases were asymmetrically grafted on one side of AuNP.

CUPJNRs show remarkable movement in biologically related fluids using the physiological concentrations of urea as a fuel. In a single fuel system, CUPJNRs showed increased motion speed with fuel concentration until reaching a plateau (Fig. 2c, Figs S10, S11, and Videos S1, S2), consistent with Michaelis−Menten enzyme kinetics. They displayed efficient propulsion at the physiological concentrations of urea in human blood (∼2.5−7.8 mM) [47], while the H_2_O_2_ concentration required to efficiently drive CUPJNRs was much higher than its physiological or pathological concentration (∼50−100 μM) in disease lesions (for example, tumor) [41]. CUPJNRs in the mixed-fuel system showed motion behaviors similar to that in the single-fuel system but had larger effective diffusion efficiency (*D*_eff_) than the latter, as seen in Fig. 2c, Fig. S12, and Video S3. This suggests the same direction of the propulsive forces arising from the two enzymatic reactions. In addition, the *D*_eff_ of CUPJNRs in the mixed-fuel system was lower than the sum of that in two different single-fuel systems. This is because the ammonia generation from urease-catalyzed decomposition of urea increases the local pH, which in turn diminishes the catalytic efficiency of catalase [48,49]. Benefitting from dense soft shells and small size at nanometer scale [50], CUPJNRs exhibited excellent ionic tolerance and could be efficiently propelled in a variety of biologically relevant media (Fig. 2d, Fig. S13, and Video S4), consistent with previously reported urease-powered nanorobots [45].

CUPJNRs in tumor microenvironments are able to actively target and accumulate in tumor cells at a much higher efficiency than previously reported nanorobots. Figure 2e shows a static microfluidic chip-based three-dimensional (3D) cell model, where the left (zone 1) and right reservoirs (zone 2) were, respectively, filled with collagen gel containing tumor cells (MCF-7) and normal cells (MCF-10A), whereas the straight channel was filled with mouse plasma. After 2 min, the nanorobots were injected into the middle reservoir (zone 3). As shown in Fig. 2f, CUPJNRs demonstrated rapidly increased absorbance in zone 4 while keeping almost unchanged in zone 5 over time. The control group (CU*PJNRs, where U* represents inactive urease, as confirmed by Fig. S14) showed no significant change in absorbance in both zones 4 and 5. This suggests that there are many more CUPJNRs targeting the collagen gel containing tumor cells than CU*PJNRs. Dark-field microscopy images showed that CUPJNRs in zone 4 exhibited a remarkable shift toward the left reservoir containing tumor cells (Fig. S15 and Video S5). At 60 min, there were 49 nanorobots in zone 6 near the reservoir containing MCF-7 and 2 nanorobots in zone 7 near the reservoir containing MCF-10A cells (Fig. 2g and Fig. S16), indicating a high selectivity to tumor cells. The H_2_O_2_ overexpressed by the tumor cells in the left reservoir forms a H_2_O_2_ concentration gradient along the straight channel of the microfluidic chip (Fig. S17). Its reaction with the catalases in CUPJNRs leads to a phoretic rotation torque over CUPJNRs, facilitating the orientation of CUPJNRs along the H_2_O_2_ concentration gradient. Thus, with the significantly accelerated propulsion from the reaction of urease with ∼10 mM urea in mouse plasma, many more CUPJNRs moved toward the left reservoir containing tumor cells, resulting in the ultrasensitive chemotaxis toward H_2_O_2_ and efficient targeting accumulation of CUPJNRs at tumor cells. As shown in Figs S18–S20 and Video S6, CUPJNRs of different sizes all showed ultrasensitive chemotaxis toward H_2_O_2_ and remarkably improved accumulation efficiency at tumor cells than the CU*PJNRs.

CUPJNRs under physiological environments of dynamic fluids also show strong tumor-targeting capability. To validate this, we designed a microfluidic chip mimicking a tumor capillary network (Fig. 2h), which featured two collagen gel reservoirs filled with tumor cells (MCF-7, zone 5) and normal cells (MCF-10A, zone 6), respectively, and a central channel perfused with mouse plasma (flow velocity: ∼300 μm/s, simulating blood flow) to connect the reservoirs. Cy5-labeled nanorobots with different formulations were injected into the channel to assess their ability to selectively accumulate in tumor regions. Figure 2i revealed that C*U*PJNRs (Passive) showed minimal adhesion with weak fluorescence confined to channel edges (<10 μm). C*UPJNRs (propulsion-only) exhibited increased penetration depth but no observable selectivity between tumor and normal zones. CU*PJNRs (orientation-only) adhered preferentially to tumor zones but failed to penetrate beyond 25 μm due to insufficient thrust. In sharp contrast, CUPJNRs exhibited a unique combination of tumor-specific accumulation (the FI in zone 5 was 12 times larger than that in zone 6) and deep penetration (>200 μm). These striking differences in their spatial distribution highlight that the synergy from urease-driven propulsion (catalytically decomposing urea) and catalase-mediated orientation (sensing H_2_O_2_ concentration gradient) endows CUPJNRs under clinically relevant hydrodynamic and biochemical conditions with significantly enhanced chemotactic sensitivity to tumors, and allows them to actively navigate against liquid flow and selectively infiltrate tumors. Thus, they hold potential for active targeting toward *in vivo* tumors with an augmented efficiency upon intravenous administration.

### Mechanism of ultrasensitive chemotaxis and enhanced targeting efficiency of CUPJNRs

Nanoparticles dispersed in a fluid generally perform random motion due to constantly being subjected to Brownian forces. Assuming that the nanoparticle is spherical, the Brownian force acting on it can be decomposed into translational Brownian force (${\mathop F\limits^{\rightarrow} }_B( t )$) and rotational Brownian torque (${\mathop M\limits^{\rightarrow} }_B( t )$). They are governed by the own Langevin equation and statistically independent, which can be, respectively, expressed as:


(1) \begin{eqnarray*}
{\mathop F\limits^{\rightarrow} }_B\left( t \right) = \ \mathop {R(t)}\limits^{\rightarrow} {F}_B,\\
{F}_B = \sqrt {\displaystyle\frac{{2{k}_BT{\zeta }_t}}{{{\mathrm{\Delta }}\tau }}} ,{\mathrm{\ }}{\zeta }_t = 6{\pi}\alpha \eta ,
\end{eqnarray*}

(2) \begin{eqnarray*}
{\mathop M\limits^{\rightarrow} }_B( t) = \ \mathop {R\left( t \right)}\limits^{\rightarrow} \ {M}_B,\\
{M}_{B = }\sqrt {\displaystyle\frac{{2{k}_BT{\zeta }_r}}{{{\mathrm{\Delta }}\tau }}} ,{\zeta }_r = 8{\pi}{\alpha }^3\eta ,
\end{eqnarray*}

where $\mathop {R( t )}\limits^{\rightarrow} $ is a random vector, of which all the components are Gaussian random numbers of zero mean and unit variance, ${k}_B$ is the Boltzmann constant (1.38 × 10^−12^ J/K), *T* is absolute temperature, $\Delta \tau $ is the time interval of action of the random force, $\alpha $ is the nanoparticle radius, $\eta $ is fluid dynamic viscosity, ${\zeta }_t$ is the translational friction coefficient, and ${\zeta }_r$ is the rotational friction coefficient. Equations (1) and (2) state that ${\mathop F\limits^{\rightarrow} }_B( t )$ and ${\mathop M\limits^{\rightarrow} }_B( t )$ are determined by the dissipative drag coefficient and the thermal energy, and balanced over time.

**Figure 3.**
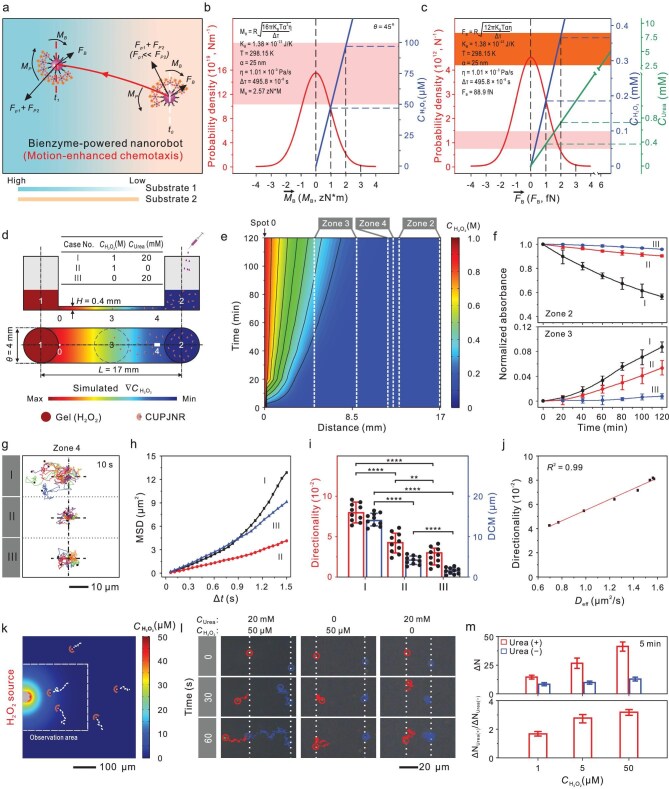
Propulsion-enhanced chemotaxis mechanism and its evidences of CUPJNRs. (a) Mechanism diagram for ultrasensitive chemotaxis of chemically propelled nanorobots. (b) Numerically simulated dependence of the alignment probability of CUPJNRs on H₂O₂ concentration. (c) Numerically simulated dependence of the probability of the chemical thrusts dominating over Brownian motion of CUPJNRs on H_2_O_2_/urea concentration. The pink and orange red areas represent the concentration of H_2_O_2_ in the tumor microenvironment and urea in the physiological environment, respectively. (d) Schematic diagram of a μ–slide microfluidic channel model. (e) 2D contour map of the H_2_O_2_ concentration in the microfluidic channel. (f) Normalized absorbance at 530 nm of CUPJNRs at zones 2 and 3 of the microfluidic channel under cases I-III [*n* = 3; mean ± SD]. (g) Typical trajectories [*n* = 5] of CUPJNRs at zone 4 of the microfluidic channel over a 10 s period in different cases, (h) corresponding mean-square-displacement (MSD) versus time interval (Δ*t*), and (i) directionality and displacement of center of mass (DCM) of CUPJNRs analyzed from the trajectories [*n* = 10; mean ± SD]. The trajectories are obtained from Videos S7 and S8, respectively. (j) Directionality versus *D*_eff_ (obtained from Fig. 2c) for CUPJNRs in a liquid of H_2_O_2_ concentration gradients containing different concentrations of urea. (k) Schematic diagram of a micropipette model to evaluate the chemotactic sensitivity of CUPJNRs. (l) Typical trajectories of CUPJNRs in different situations (Video S9). (m) The increased number (Δ*N*) and ratio of the CUPJNRs within the dashed box in (k) with or without 20 mM urea versus the H_2_O_2_ concentrations filled in the micropipette over 5 min [*n* = 3; mean ± SD]. The Δ*N* of nanorobots within the dashed box after different treatments is shown in Figs S25−S27. Statistical comparisons were tested using an ordinary one-way analysis of variance (ANOVA) with Tukey’s multiple-comparisons test. ****P < 0.0001; **P < 0.01.

As illustrated in Fig. 3a, for chemically propelled nanorobots in a chemoattractant gradient to achieve strong chemotactic movement, they must simultaneously generate both a torque (*M_p_*, tangential to the radius) to counterbalance the fluctuation of ${\mathop M\limits^{\rightarrow} }_B( t )$ and a sufficient strong chemical propulsion (*F_p_*, with a direction along the symmetric axis for Janus nanorobots) to overcome the fluctuation of ${\mathop F\limits^{\rightarrow} }_B( t )$. *M_p_* works for the orientation of nanorobots along the chemoattractant gradient, and *F_p_* guarantees directional movement parallel to the symmetric axis of the nanorobots. We call it a propulsion-enhanced chemotaxis mechanism. Figure S21a displays that only with strong *F_p_* to counteract ${\mathop F\limits^{\rightarrow} }_B( t )$, they perform enhanced diffusion without directional bias due to the random ${\mathop M\limits^{\rightarrow} }_B( t )$. The strong ${\alpha }^3$ dependence of ${\zeta }_r$ versus $\alpha $ dependence of ${\zeta }_t$ in Equations (1) and (2) indicates that rotational diffusion is much more sensitive to $\alpha $ than translational diffusion. When $\alpha $ reduces to the nanometer scale, ${\zeta }_t$ is much larger than ${\zeta }_r$, suggesting that the force required to overwhelm ${\mathop F\limits^{\rightarrow} }_B( t )$ is much greater than that required to overwhelm ${\mathop M\limits^{\rightarrow} }_B( t )$. Chemically powered micro-/nanorobots in a fuel concentration gradient can generate *M*_p_ to align along the gradient, resulting in chemotaxis [40]. However, in a fluid with an enough chemoattractant gradient to generate powerful *M_p_* to counteract ${\mathop M\limits^{\rightarrow} }_B( t )$ but a too low fuel concentration to produce strong *F_p_* to counteract ${\mathop F\limits^{\rightarrow} }_B( t )$, they can orientate themselves along the gradient but move without obvious directionality, resulting in minimal chemotactic displacement (Fig. S21b).

For CUPJNRs ($2\alpha$= 50 nm) in a fluid with $\eta$= 1.01 × 10^−3^ Pa/s at 298.15 K, $\Delta \tau $ was determined to be ∼495.8 μs from DSL analysis, and substituting them into equations (1) and (2), respectively, yields *F_B_* = 88.9 fN and *M_B_* = 2.57 zN*m. It is assumed that the angle between the symmetric axis of CUPJNR and the direction of the H_2_O_2_ concentration gradient is 45^o^. As shown by the numerical simulations in Fig. 3b, the gradient produced by a local source of 43 μM H_2_O_2_ allows CUPJNRs to generate *M_p_* comparable to *M_B_*. Correspondingly, the probability of CUPJNRs to align along the chemoattractant H_2_O_2_ gradient reaches 68%. However, CUPJNRs using H_2_O_2_ as a fuel needs more than 190 μM H_2_O_2_ to get *F_p_* close to *F_B_* (Fig. 3c). That is to say, the physiologic 50–100 μM H_2_O_2_ typically appearing in tumor microenvironments is sufficient to enable 68% CUPJNRs to induce directional bias, but too low to generate translational propulsion. Figure 2c indicates that notably moving CUPJNRs requires >10 mM H_2_O_2_. This explains the weak chemotactic behavior of previously reported single-enzyme chemotactic nanorobots in physiological environments. With physiological 2.5–7.8 mM urea, CUPJNRs produce *F_p_* much more than *F_B_* via the urease–urea reaction (Fig. 3c), besides the *M_p_* (comparable to *M_B_*) generated by the catalase reaction with physiological H_2_O_2_ gradient, enables the alignment of most CUPJNRs along the H_2_O_2_ gradient. Thus, they possess ultrasensitive chemotactic behavior toward tumor cells in tumor microenvironments. This suggests that the decoupling of orientation and propulsion with chemoattractant and fuel endows CUPJNRs with a markedly enhanced chemotactic capability.

To further prove and decipher the above proposed propulsion-enhanced chemotaxis mechanism of CUPJNRs, we systematically investigated the roles of propulsion (urease/urea) and orientation (catalase/H_2_O_2_) in the enhancement of the chemotactic sensitivity and targeting efficiency of CUPJNRs using a *μ*-slide microfluidic chemotaxis model (Fig. 3d). In this model, the left reservoir (zone 1) was loaded with agarose gel containing H_2_O_2_ aqueous solution to establish a H_2_O_2_ concentration gradient along the straight channel filled with urea aqueous solutions. H_2_O_2_ can fill the entire channel within 20 min, as verified using a H_2_O_2_ fluorescent probe (Fig. S22). The 2D contour map in Fig. 3e showed the H_2_O_2_ concentration gradients formed in the microfluidic channel over a 2-h period. CUPJNRs were injected into the right reservoir (zone 2). The collective chemotactic behaviors of CUPJNRs under the three conditions listed in the inset table in Fig. 3d are starkly different, as shown by their corresponding quantitative absorbance profiling in Fig. 3f. For the experimental group (case I), CUPJNRs migrated robustly toward the H_2_O_2_ source (zone 1), with a 42% depletion in zone 2 and progressive accumulation in zone 3 over 2 h. In contrast, for case II, in the same H_2_O_2_ gradient but in the absence of urea, CUPJNRs were confined in zone 2 with a small decrease (∼9.5%) after 2 h, only marginally higher than passive diffusion (∼5%). CUPJNRs without propulsion have difficulty in exhibiting efficient chemotaxis even when the concentration of chemotactic source (H_2_O_2_) is as high as 1 M in zone 1. In case III, CUPJNRs in urea yielded rapid but undirected diffusion, confirming that without H_2_O_2_ gradient they fail to perform targeted accumulation. These results are consistent with the prediction of the propulsion-enhanced chemotaxis mechanism.

High-resolution tracking of individual CUPJNRs in zone 4 by an inverted dark-field microscope (Fig. 3g and h) further illustrated these mechanisms. CUPJNRs in case I exhibited directional trajectories (Fig. 3g and Videos S7, S8) with near-linear mean-squared displacement (MSD) curves (Fig. 3h), indicative of significantly enhanced ballistic motion toward zone 1. In contrast, CUPJNRs in case II exhibited slow, biased movement toward zone 1, while those in case III displayed rapid but random mobility. Figure 3i further decoupled the motion behavior of CUPJNRs into directionality and displacement of center of mass (DCM), both of which significantly decreases following the order of case I > case II > case III, underscoring the necessity of urease/urea reaction-driven propulsion to amplify chemotactic behavior of CUPJNRs induced by catalase/H_2_O_2_ gradient reaction, suggesting propulsion-enhanced chemotactic sensitivity.

The chemotactic efficacy of CUPJNRs to H_2_O_2_ scaled linearly with the concentration of urea (Fig. S23). For CUPJNRs in a physiologically attainable concentration of 20 mM urea and H_2_O_2_ concentration gradients, the *D*_eff_, directionality, and DCM values reached over 2.0 μm^2^/s, 0.06 and 15 μm, respectively, enabling navigation in liquid flow. Importantly, the positive linear correlation of the directionality with *D*_eff_ (Fig. 3j) confirms that propulsion force (*F*_p_) directly enhances chemotactic precision against Brownian motion (*R*^2^ = 0.99).

The exceptional targeting efficiency of CUPJNRs hinges on their ability to sense tumor-relevant H_2_O_2_ concentration gradients. To quantify this sensitivity [51], we carried out a micropipette experiment (Fig. 3k and Fig. S24), where a controlled H_2_O_2_ concentration gradient was generated in a physiological urea solution. As predicted in the propulsion-enhanced chemotaxis mechanism, at 50 μM H_2_O_2_, which closely mimics the tumor microenvironment, CUPJNRs exhibited a robust chemotactic shift toward the micropipette, accumulating within the detection zone at a rate 3 times higher than that of the control group lacking urea (Fig. 3l, m, and Video S9). Even at an H_2_O_2_ concentration as low as 1 μM, nearly 50-fold lower than typical physiological levels, CUPJNRs with urea fuel still maintained obvious chemotactic movement (Fig. 3m and Figs S25−S27). This validates that urease/urea reaction-driven propulsion significantly amplifies the chemotactic navigation capability at previously inaccessible chemoattractant levels. In addition, CUPJNRs in mixed-fuel systems exhibit improved chemotactic sensitivity but decreased catalase activity by ∼48% (Fig. S28), compared to those in single H_2_O_2_. This also implies that the improved chemotactic sensitivity stems from the coupling of propulsion and orientation rather than from catalytic enhancement.

To further validate the exclusive role of ureases in propulsion, we designed nanorobots with catalases and ureases on opposite hemispheres (Fig. S29). In H_2_O_2_ gradients without urea, they weakly migrated toward the source due to catalase-generated faint *F_p_*. With urea present, they reversed direction due to urease-generated thrust overpowering *F_B_* (Fig. S29 and Video S10). This inversion rigidly confirms the decoupling of propulsion and orientation. Additionally, replacing catalase with glucose oxidase (GUPJNRs) yielded identical chemotactic enhancement toward glucose gradients in the presence of urea (Figs S30−S32 and Videos S11, S12). The linear *D*_eff_-directionality correlation persisted (Fig. S32), proving that propulsion augmentation is a universal strategy to enhance chemotactic sensitivity.

### *In vivo* tumor targeting accumulation and penetration of CUPJNRs

**Figure 4.**
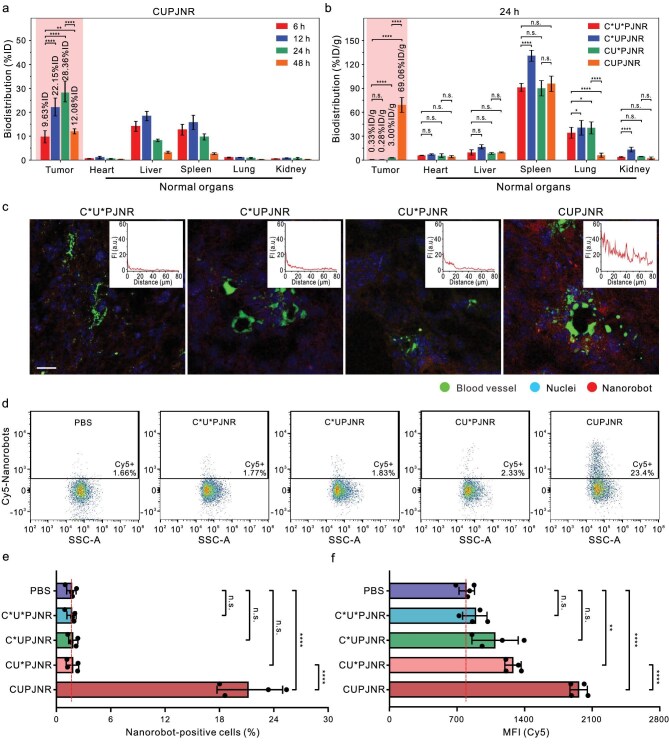
*In vivo* tumor targeting accumulation, penetration, and cellular internalization of CUPJNRs. (a) Biodistribution of CUPJNRs (90 nm) in the tumor-bearing mice model at different time points after intravenous injection of 1.36 billion CUPJNRs [*n* = 4; mean ± SD]. (b) Biodistribution of the nanorobots with different formulations in the tumor-bearing mice model at 24 h after intravenous injection of 1.36 billion nanorobots [*n* = 4; mean ± SD]. (c) Representative confocal fluorescence images of resected tumors from different groups of mice. The nanorobots were modified with Cy5 (red), and the nuclei were stained with Hoechst 33342 (blue), and the blood vessels were stained with DyLight 488-modified goat anti-mouse IgG (green). Scale bar: 20 μm. Insets were the plots of mean FI (MFI) of Cy5 versus the distance from the nearest blood vessel [*n* = 4]. (d) Representative flow cytometry images of nanorobot-positive CD45-negative tumor-associated cells in tumors from different groups of mice. (e) Percentage of nanorobot-positive CD45-negative tumor-associated cells and (f) Mean FI (MFI) of Cy5 within nanorobot-positive CD45-negative tumor-associated cells [*n* = 4; mean ± SD]. The vertical dashed lines indicated the background signals. Statistical comparisons were tested using an ordinary one-way ANOVA with Tukey’s multiple-comparisons test. ****P < 0.0001; **P < 0.01; *P < 0.05; n.s., not significant.

With ultrasensitive chemotaxis toward H_2_O_2_, CUPJNRs injected into tumor-bearing mice via tail veins can *in vivo* actively search for tumors and demonstrate a high targeting efficiency at disease sites. The tumor-bearing mice, which were constructed following [16], were euthanized at different time points after intravenous injection, and major organs were harvested for investigating the biodistribution of CUPJNRs *in vivo*. As revealed by ICP−MS analysis in Fig. 4a and Fig. S33, the delivery efficiency of CUPJNRs at tumors was much higher than that in normal organs. After 24 h intravenous injection of 1 billion CUPJNRs, their *Ɛ* at tumors reached a peak value as high as ∼28.36%ID, corresponding to ∼69.06%ID/g. The biodistribution of the nanorobots with different formulations at 24 h after intravenous administration are compared in Fig. 4b, which illustrates that CUPJNRs showed the highest *Ɛ* at tumors. Their *Ɛ* was much higher than that of the passive nanoparticles (C*U*PJNRs), urease-powered nanorobots (C*UPJNRs), and single fuel-chemotactic nanorobots (CU*PJNRs) by 209 times, 246 times, and 23 times, respectively. This can be explained by the fact that there is a persistent higher concentration of H_2_O_2_ in tumors than that of normal tissues, as confirmed in Fig. S34. CUPJNRs perform ultrasensitive chemotaxis in higher concentrations of H_2_O_2_, resulting in their superhigh *Ɛ* at tumors. C*U*PJNRs demonstrate a comparable tumor-targeting efficiency to that of AuNPs at the same ID [15]. CU*PJNRs exhibit higher tumor-targeting efficiency by one order of magnitude (9.8-fold) than C*U*PJNRs, consistent with the previously reported study [16]. Thus, the as-developed nanorobots have demonstrated significant improvement in both absolute tumor-targeting efficiency and the multiple of increased targeting efficiency relative to their passively diffused counterparts.

Notably, the accumulation of CUPJNRs in the liver and spleen was comparable to that of the control groups, suggesting that they did not exhibit an enhanced ability to evade macrophage-mediated clearance in these organs. In contrast, their accumulation in the heart, lung, and kidney was markedly lower than that of the control groups. Both C*UPJNRs and CUPJNRs demonstrated good motility under physiological environments. Among them, C*UPJNRs showed increased accumulation in the spleen compared with other formulations, whereas CUPJNRs displayed a distribution pattern similar to that of C*U*PJNRs. These results collectively suggest that the significantly enhanced active tumor-targeting capability of CUPJNRs accounts for their preferential accumulation at tumor sites rather than in normal organs. Furthermore, CUPJNRs demonstrated a distinct kinetic profile in tumors and normal organs. The level of CUPJNRs in tumors continuously increased for 24 h, while that in normal organs showed a peak at 12 h. The longer retention time of CUPJNRs inside tumors could be attributed to the positive chemotaxis of nanorobots, which disrupts the balance between their extravasation and intravasation through tumor blood vessels. CUPJNRs can maintain good motion stability, and the *D*_eff_ only decreased 3% after incubation in DMEM for 48 h (Fig. S35). As shown in Fig. S36, the targeting efficiency of CUPJNRs in tumor tissue reached 30.52%ID (71.88%ID/g) at 24 h after intravenous injection of 5 billion nanorobots (1.835 mg/kg). This indicates that the tumor-targeting efficiency of CUPJNRs can be further improved by increasing their ID if it is within the safe tolerance of bodies, in accordance with the literature reported by Chan *et al.* [15]. These above results collectively suggest that the high *Ɛ* of CUPJNRs at tumors positively correlate with their ultrasensitive chemotactic capability.

CUPJNRs after extravasating blood vessels can penetrate deeply into tumors and efficiently internalize into tumor cells. Figure 4c shows that the C*U*PJNR (corresponding to passive AuNPs), C*UPJNRs (urease-powered nanorobots), and CU*PJNRs (single-fuel chemotactic nanorobots) were predominantly co-localized with blood vessels (green), whereas CUPJNRs diffused far away from vasculature and exhibited a more uniform distribution throughout the tumors. As presented in the upper right inset, the fluorescence intensity (FI) of C*U*PJNRs, C*UPJNRs, and CU*PJNRs groups decayed rapidly with distance, and the signal was hardly detectable after 15 μm, whereas CUPJNRs were uniformly distributed around the blood vessels and penetrated more than 80 μm. The cumulative FI of Cy5 at distances between 0 and 100 μm from the blood vessel in various directions showed that the penetration capability of CUPJNRs was more than 12 times that of C*U*PJNRs (Fig. S37). Figure S38 demonstrates that the enhanced penetration capability of CUPJNR within tumors was mainly derived from the propulsive force generated by the urease and urea reaction system [52].

The flow cytometry analysis results in Fig. 4d, e, and Fig. S39 demonstrated that C*U*PJNRs, C*UPJNRs, and CU*PJNRs displayed no obvious difference in the internalization capability of CD45-negative tumor-associated cells. After deducting the background signal shown in the PBS-treated group, there were only ∼0.01%, 0.19%, and 1.64% of CD45-negative tumor-associated cells that contain nanorobots for the groups of C*U*PJNRs, CU*PJNRs, and C*UPJNRs, respectively. The percentage of nanoparticle-positive CD45-negative tumor-associated cells for the control group (C*U*PJNR) was comparable to that of AuNPs reported previously [11,15], suggesting a reliable result. In contrast, for the CUPJNRs group, >19.70% of CD45-negative tumor-associated cells absorbed nanorobots, which was 1970-fold and 103-fold higher than that in the groups of C*U*PJNRs and CU*PJNRs. Fig. 4f indicates that the amount of CUPJNRs in CD45-negative tumor-associated cells also increased significantly compared with the group of C*U*PJNRs, attaining high internalization efficiency in CD45-negative tumor-associated cells even at a remarkably low injection dose.

In addition, the cellular uptake behavior of CUPJNRs was also explored at different conditions (Fig. S40). The FI of the CUPJNRs group was substantially higher than that of the C*U*PJNRs group, indicating that motion behavior can significantly enhance the cellular uptake of nanorobots. For incubation at 4°C, the CUPJNRs group showed almost no fluorescence, indicating that the cellular uptake of CUPJNRs was almost completely inhibited. This suggests that their internalization is energy-dependent. Hypertonic sucrose inhibited 75% of cellular uptake, while the other two inhibitors had less noticeable effects. Considering that hypertonic sucrose is a typical clathrin inhibitor, it suggests that CUPJNRs are primarily internalized through clathrin-mediated endocytosis.

Taken together, after being intravenously injected, CUPJNRs can perform robust chemotactic movement, and significantly increase the efficiency to break through the major biological barriers of a five-step ‘CAPIR’ cascade [44], including accumulation at solid tumor, penetration into avascular tumor tissue, and cell internalization, thus deeply penetrating the solid tumor to reach distal cancer cells.

### *In vivo* antitumor efficacy of Ce6-CUPJNRs

**Figure 5.**
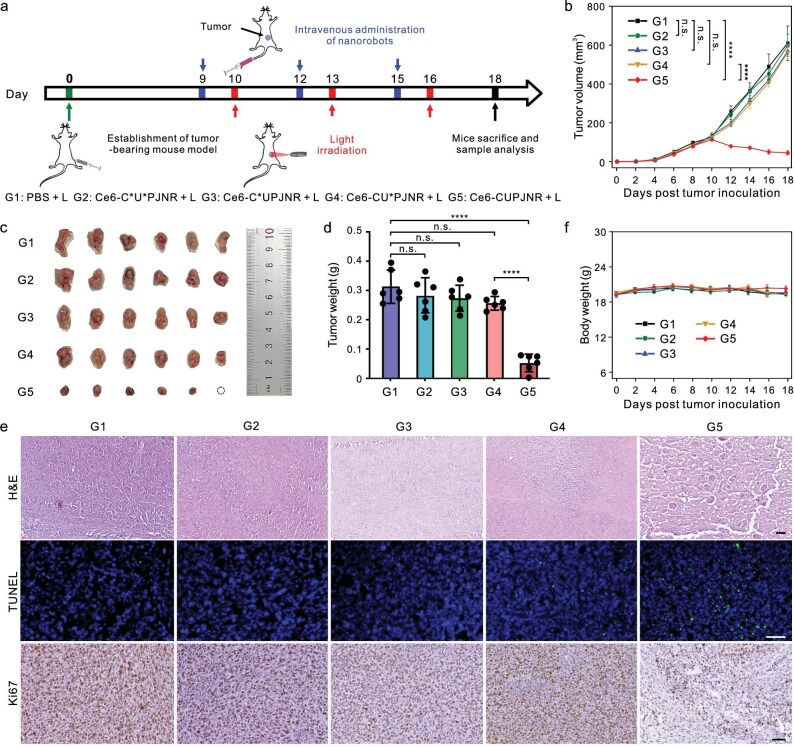
Antitumor efficacy of Ce6-CUPJNRs *in vivo*. (a) Schematic of the mouse model that bears subcutaneous 4T1 tumor and the following treatment protocol. Twenty-four hours after injection of PBS or nanorobots with different formulations (Ce6, 3 μg/kg) every 3 d for a total of 3 doses, the tumor sites were exposed to 660 nm laser irradiation at a power density of 0.18 W/cm^2^ for 10 min. The nanorobots were modified with photosensitizers (Ce6). (b) Tumor growth kinetics of tumor-bearing mice with indicated treatments [*n* = 6; mean ± standard error of mean (SE)]. (c) Images of tumors from mice at the end of treatment. (d) Excised tumor weights from mice at the end of treatment [*n* = 6; mean ± SD]. (e) Representative images of H&E, TUNEL, and Ki67 staining of resected tumors from mice from different groups. Scale bars: 100 μm. (f) Body weight of mice with different treatments over the treatment process [*n* = 6; mean ± SE]. Statistical comparisons in (b) and (d) were tested using an ordinary one-way ANOVA with Tukey’s multiple-comparisons test. ****P < 0.0001; n.s., not significant.

To further investigate the antitumor efficacy of CUPJNRs as drug delivery carriers, a photodynamic PDT delivery system (denoted as Ce6-CUPJNRs) was established by modifying an FDA-approved photosensitizer Ce6 (FDA UNII: 5S2CCF3T1Z, a widely used antitumor drug for photodynamic therapy) on the surface of CUPJNRs (Figs S41−S43). As schematically illustrated in Fig. 5a, a 4T1 tumor-bearing mouse model was created by direct subcutaneous implantation of nearly 2 × 10^7^ 4T1 tumor cells per BALB/c mouse. When the tumor volume reached ∼100 mm^3^, different nanorobots containing Ce6 (3 μg/kg) were intravenously injected into the mouse tail vein every 3 d for a total of 3 doses. Twenty-four hours after injection, the tumor sites were exposed to 660 nm laser irradiation at a power density of 0.18 W/cm^2^ for 10 min. The tumor volumes were recorded every 2 d (Fig. 5b and Fig. S44). Similar to the PBS-treated group, the tumor volumes increased rapidly in both Ce6-C*U*PJNRs, Ce6-C*UPJNRs, and Ce6-CU*PJNRs-treated groups, which suggests a negligible tumor suppression effect. In contrast, the Ce6-CUPJNRs-treated group showed a significant reduction in the tumor volume after the first dose. The tumor volume was finally reduced to ∼44.3 mm^3^ after the treatment, with a 92.7% TGI rate. Compared with the passive counterparts, CUPJNRs boosted the tumor suppression efficacy of the drugs by about 49 times. As indicated in Table S2, the nanorobots achieved a comparable therapeutic efficacy only with an ID <1% of the dosage of previously reported drug delivery systems, indicating high bioavailability (Fig. S45). Figure 5c and d further confirmed that Ce6-CUPJNRs efficiently inhibited the growth of tumors in size and weight. The results of H&E staining, TUNEL assays, and immunohistochemistry analysis (Fig. 5e) for tumors revealed a distinctive feature such as cell shrinkage, increased cell apoptosis, and a marked reduction in cell proliferation were evident after treatment with Ce6-CUPJNRs. The significantly improved therapeutic efficacy of Ce6-CUPJNRs can be attributed to the synergistic effect of high tumor-targeting delivery efficiency, strong tumor penetration, and tumor cell internalization capabilities of the nanorobots.

Last, we evaluated the *in vivo* safety profile of mice after treatment with Ce6-CUPJNRs. The treated mice showed no notable loss in body weight during the treatment (Fig. 5f), suggesting that the administration of Ce6-CUPJNRs was well-tolerated by the mice. In addition, a comprehensive blood chemistry panel was conducted on the 18th day. Like untreated control mice, the mice receiving the Ce6-CUPJNRs treatment also showed normal levels of all serum biochemistry markers (Fig. S46). Furthermore, H&E staining of the major organ tissue sections did not show obvious organ damage compared with the normal group (Fig. S47). These results collectively indicate that the nanorobots possess good *in vivo* biosafety.

## CONCLUSIONS

In this work, we demonstrated an effective strategy for chemically propelled nanorobots to perform ultrasensitive chemotactic mobility by designing CUPJNRs, where two distinct enzymes (urease and catalase) are chemically linked to the same semi-surfaces of AuNPs, and serve for the propulsion and orientation functionalities, respectively. CUPJNRs, as an anti-tumor drug nanocarrier, have addressed the long-standing critical issues of low *Ɛ* and poor penetration capability of nanoparticle-based drug delivery systems. In a simulated tumor microenvironment, they are able to produce a torque to counterbalance the fluctuation of ${\mathop M\limits^{\rightarrow} }_B( t )$ by leveraging the pathological concentration gradient of 50–100 μM H_2_O_2_, enabling orientation along the chemoattractant gradient, and a sufficiently strong propulsion force to overcome the fluctuation of ${\mathop F\limits^{\rightarrow} }_B( t )$ by leveraging endogenous urea (2.5–7.8 mM), guaranteeing directional movement parallel to the symmetric axis of nanorobots. Thus, they show ultrasensitive chemotaxis toward tumor cells. In a tumor-bearing mouse model, they exhibited markedly improved tumor-targeting efficiency, penetration depth, and cellular internalization compared with their passive counterparts. When loaded with the photosensitizer Ce6 for photodynamic therapy, they achieved a tumor growth inhibition rate of 92.7% with an ID reducing to 1% of previous delivery systems and amplified the therapeutic efficacy by 49-fold, underscoring their potential for precise and efficient drug delivery. To facilitate the clinical translation of CUPJNRs, we plan to develop scalable fabrication methods, establish a comprehensive preclinical safety profile, and assess their efficacy and safety in large animal models (e.g. beagles). This reported propulsion-enhanced chemotaxis strategy is applicable for different application scenarios of chemotactic nanorobots by changing the combination of chemical reaction systems. This work holds promise for the design of intelligent nanorobots in the context of active targeting of drug delivery and precision medicine in the foreseeable future.

## Supplementary Material

nwaf580_Supplemental_Files
