# Supplementary material for: Bienzyme-powered nanorobots with ultrasensitive chemotaxis for precision cancer therapy
Source: Natl Sci Rev. 2025 Dec 18;13(3):nwaf580. doi: 10.1093/nsr/nwaf580 (PMC12866669; doi:10.1093/nsr/nwaf580)
Supplement: nwaf580_Supplemental_Files [file nwaf580_supplemental_files.zip › Supplementary data.pdf]

Supporting Information for

Bienzyme-Powered Nanorobots with Ultrasensitive Chemotaxis for Precision Cancer Therapy

Zili Yang[1,#], Ziye Pei[1,#], Zhixue Gao[1,#], Ming Luo[1,2,*], Xingchi Liu[3,4], Jie Guo[1], Huanyu Jiang[1], Mengting Lv[1], Zili Yu[3,4,*], Suling Zhao[5] and Jianguo Guan[1,2,*]

[1]State Key Laboratory of Advanced Technology for Materials Synthesis and Processing, International School of Materials Science and Engineering, Wuhan University of Technology, Wuhan 430070, China

[2]Wuhan CyneMed Technology Co., Ltd, Wuhan 430207, China

[3]State Key Laboratory of Oral and Maxillofacial Reconstruction and Regeneration, Key Laboratory of Oral Biomedicine Ministry of Education, Hubei Key Laboratory of Stomatology, School & Hospital of Stomatology, Wuhan University, Wuhan 430079, China

[4]Department of Oral and Maxillofacial Surgery, School and Hospital of Stomatology, Wuhan University, Wuhan 430079, China

[5]Center for Materials Research and Analysis, Wuhan University of Technology, Wuhan 430070, China

***Corresponding authors**. E-mails: luoming_2016@whut.edu.cn; zili09@whu.edu.cn; guanjg@whut.edu.cn

[#]Equally contributed to this work.

Table of Contents

1. Experimental methods……………………………………………………………………………S6

Materials

Synthesis of 50 and 90 nm AuNPs

Synthesis of EAPNPs

Synthesis of biotin-modified Janus AuNPs

Synthesis of biotinylated enzyme molecules

Preparation of CUPJNRs

Motion behavior of CUPJNRs

In vitro tumor-targeting behavior of CUPJNRs

Quantification of urease activity

Quantification of catalase activity

Chemotaxis of CUPJNRs

Dynamic chemotactic behavior of CUPJNRs

Numerical simulation

Preparation of the nanorobots with catalases and ureases asymmetrically modified on the two opposite sides of AuNPs

Chemotactic behavior of the nanorobots with catalases and ureases asymmetrically modified on the two opposite sides of AuNPs

In vivo biodistribution measurement of CUPJNRs

Detection of H_2O_2 content in tissue

Synthesis of Cy5-modified CUPJNRs and FITC-modified CUPJNRs

Penetration capability of CUPJNRs

Intratumoral penetration capability of CUPJNRs

Internalization of CUPJNRs by tumor-associated cells and flow cytometry

Cellular uptake of the CUPJNRs

Preparation of Ce6-loaded CUPJNRs (Ce6-CUPJNRs)

In vivo antitumor efficacy of Ce6-CUPJNRs

In vivo antitumor efficacy of Ce6

Instruments

Statistical analysis

2. Supplementary Figures..S17

Supplementary Figure 1. Schematic illustration of the preparation procedure used to fabricate the CUPJNRs.

Supplementary Figure 2. TEM images and UV-Vis absorption spectrum of 50 nm AuNPs.

Supplementary Figure 3. SEM and TEM images of the EAPNPs.

Supplementary Figure 4. SEM and TEM images of the partially etched EAPNPs by THF.

Supplementary Figure 5. UV-Vis absorption spectrum of 50 nm CUPJNRs.

Supplementary Figure 6. The loading amounts the catalase and urease on 50 nm CUPJNRs.

Supplementary Figure 7. Hydrodynamic size of the products during the preparation of the 50 nm CUPJNRs.

Supplementary Figure 8. The 2D morphology images and height profile distribution of the products during the preparation of the 50 nm CUPJNRs.

Supplementary Figure 9. The TEM images of SA-QDs asymmetrically assembled CUPJNRs, PEG-modified AuNP and CUPJNR.

Supplementary Figure 10. Motion behavior of the 50 nm CUPJNRs in urea aqueous solution.

Supplementary Figure 11. Motion behavior of the 50 nm CUPJNRs in H_2O_2 aqueous solution.

Supplementary Figure 12. Motion behavior of the 50 nm CUPJNRs in the mixed solution of urea and H_2O_2.

Supplementary Figure 13. Motion behavior of the 50 nm CUPJNRs in different biological media.

Supplementary Figure 14. The activity of urease before and after heating.

Supplementary Figure 15. Individual chemotactic behavior of the 50 nm CUPJNRs or CU*PJNRs in 3D cell model.

Supplementary Figure 16. Images of zones 6 and 7 of the microfluidic channel at different time points.

Supplementary Figure 17. Representative fluorescence images of H_2O_2 fluorescent probes at different positions in the microfluidic channel overtime.

Supplementary Figure 18. Absorbance of the 90 nm CUPJNRs or CU*PJNRs at zones 3−5 of the microfluidic channel within 60 minutes in different situations.

Supplementary Figure 19. Individual chemotactic behavior of the 90 nm CUPJNRs or CU*PJNRs in 3D cell model.

Supplementary Figure 20. Images of zones 6 and 7 of the microfluidic channel at different time points.

Supplementary Figure 21. Propulsion-enhanced chemotaxis mechanism for single fuel-powered nanorobots and single enzyme-chemotactic nanorobots.

Supplementary Figure 22. Representative fluorescence images for H_2O_2 fluorescent probes at different positions in the μ–slide microfluidic channel overtime.

Supplementary Figure 23. Chemotactic behavior of the 50 nm CUPJNRs in a H_2O_2 concentration gradient containing different concentrations of urea.

Supplementary Figure 24. Image of the device for evaluating the chemotactic sensitivity of CUPJNRs.

Supplementary Figure 25. The increased number of the 50 nm CUPJNRs in the observation region around the micropipette when the micropipette was filled with 50 μM H_2O_2 within 5 min.

Supplementary Figure 26. The increased number of the 50 nm CUPJNRs in the observation region around the micropipette when the micropipette was filled with 5 μM H_2O_2 within 5 min.

Supplementary Figure 27. The increased number of the 50 nm CUPJNRs in the observation region around the micropipette when the micropipette was filled with 1 μM H_2O_2 within 5 min.

Supplementary Figure 28. The catalytic activity of catalase and urease immobilized on the surface of 50 nm CUPJNR in a single-fuel or mixed-fuel system.

Supplementary Figure 29. Chemotactic behavior of the nanorobots with catalases and ureases asymmetrically modified on the two opposite sides of 50 nm AuNPs.

Supplementary Figure 30. Characterization of the 50 nm glucose oxidase/urease-powered Janus nanorobots.

Supplementary Figure 31. Chemotactic behavior of the 50 nm GUPJNRs in a *μ*–slide microfluidic channel.

Supplementary Figure 32. Chemotactic behavior of the 50 nm GUPJNRs in a glucose concentration gradient containing different concentrations of urea.

Supplementary Figure 33. *In vivo* targeting accumulation and penetration of the 90 nm CUPJNRs.

Supplementary Figure 34. Representative H_2O_2 fluorescence staining images of lung and tumor.

Supplementary Figure 35. The stability of CUPJNR in physiological media.

Supplementary Figure 36. Tumor-targeting efficiency of the 90 nm C*U*PJNR and CUPJNR in the tumor-bearing mouse model.

Supplementary Figure 37. The cumulative fluorescence intensity of Cy5 from the blood vessel.

Supplementary Figure 38. The intratumoral penetration capability of the 90 nm CUPJNRs with different formulations *in vivo*.

Supplementary Figure 39. Flow cytometry gating strategy for investigating cells that took up the 90 nm CUPJNRs.

Supplementary Figure 40. Cellular uptake of the 90 nm C*U*PJNRs and CUPJNRs.

Supplementary Figure 41. Preparation schematic illustration and characterization of the 90 nm Ce6-CUPJNRs.

Supplementary Figure 42. The loading amount of Ce6 on the surface of 90 nm CUPJNRs.

Supplementary Figure 43. The photostability of CUPJNR in physiological media.

Supplementary Figure 44. Individual tumor growth curves after different treatments.

Supplementary Figure 45. *In vivo* antitumor efficacy of Ce6.

Supplementary Figure 46. Blood chemistry panel of mice non-treated or treated with the Ce6-CUPJNR.

Supplementary Figure 47. Representative photographs of H&E-stained histological sections of major organs from mice non-treated or treated with the Ce6-CUPJNR.

3. Supporting Tables……………………………………………………………………………S65

Supplementary Table 1. Summary of the synthetic chemotactic MNMs.

Supplementary Table 2. Summary of the Ce6-based nanocarrier delivery systems for tumor photodynamic therapy.

4. Supporting Videos

Supplementary Video 1. Motion behavior of the 50 nm CUPJNRs in urea aqueous solution.

Supplementary Video 2. Motion behavior of the 50 nm CUPJNRs in H_2O_2 aqueous solution.

Supplementary Video 3. Motion behavior of the 50 nm CUPJNRs in the mixed solution of urea and H_2O_2.

Supplementary Video 4. Motion behavior of the 50 nm CUPJNRs in different biological media.

Supplementary Video 5. Chemotactic behavior of the 50 nm CUPJNRs and CU*PJNRs in 3D cell model.

Supplementary Video 6. Chemotactic behavior of the 90 nm CUPJNRs and CU*PJNRs in 3D cell model.

Supplementary Video 7. Chemotactic behavior of the 50 nm CUPJNRs in urea aqueous solution at zone 4 of the microfluidic channel.

Supplementary Video 8. Chemotactic behavior of the 50 nm CUPJNRs in a H_2O_2 concentration gradient containing different concentrations of urea.

Supplementary Video 9. Chemotactic behavior of the 50 nm CUPJNRs when the micropipette was filled with 50 μM H_2O_2.

Supplementary Video 10. Chemotactic behavior of the nanorobots with catalases and ureases asymmetrically modified on the two opposite sides of 50 nm AuNPs in urea aqueous solution at zone 4 of the microfluidic channel.

Supplementary Video 11. Chemotactic behavior of the 50 nm GUPJNRs in urea aqueous solution at zone 4 of the microfluidic channel.

Supplementary Video 12. Chemotactic behavior of the 50 nm GUPJNRs in a glucose concentration gradient containing different concentrations of urea.

Experimental methods

Materials: $HAuCl_4\cdot 3H_2O$ (99.99%), 4-styrenesulfonic acid sodium salt hydrate (NaSS), trisodium citrate (99%), styrene (≥ 99%), potassium persulphate (KPS, 99.99%), divinylbenzene (DVB, 80%), sodium nitroprusside, sodium hypochlorite (4.00–4.99%), 1-(3-Dimethylaminopropyl)-3-ethylcarbodiimide hydrochloride (EDC, 98%), sodium salicylate (≥99.5%), N-Hydroxy succinimide (NHS, 98%), urease, urea (99.0–100.5%), catalase, H_2O_2 (30%), glucose oxidase, glucose (≥ 99%), streptavidin (SA), Hoechst 33342, collagenase IV, cytochalasin D and sucrose were purchased from Sigma-Aldrich (Saint Louis, USA). HS-PEG-OCH_3, HS-PEG-Biotin, N-Succinimidyl-6-Biotinamidohexanoate and collagen were bought from Aladdin Biochemical Technology Co., Ltd (Shanghai, China). HOOC-PEG-SH were bought from Aladdin Biochemical Technology Co., Ltd (Shanghai, China). SA-QDs were received from Wuhan Jiayuan Quantum Dots Co., Ltd (Wuhan, China). Catalase activity assay kit was purchased from Elabscience Biotechnology Co., Ltd (Wuhan, China). Fetal bovine serum (FBS) was received from Biological Industries (Israel). Agarose (≥ 99%) was bought from Biowest (Loire Valley, France). MCF-7 cells, MCF-10A cells, and B16F10 cells were bought from Procell Life Science & Technology Co., Ltd (Wuhan, China). The Cell Count Kit-8 (CCK8) was purchased from LABLEAD.Inc. (Beijing, China). Plasmocin Treatment was bought from Beijing Solarbio Life Science& Technology Co., Ltd. (Beijing, China). Mice were purchased from Hunan Slake Jingda Laboratory Animal Co., Ltd (Changsha, China). Cyanine5 NHS ester (Cy5-NHS ester) was bought from Xi'an Qiyue Biotechnology Co., Ltd. (Xian, Chian). 5/6FAM-NHS was bought from Xinweichuang Biology (Chongqing, China). Matrigel was bought from Yeasen Biotech Co., Ltd. (Shanghai, China). APC/Cyanine7 anti-mouse CD45 antibody was bought from BioLegend (San Diego, USA). CD34 monoclonal antibody (RAM34) and live/dead fixable aqua dead cell stain kit were bought from Thermo Scientific (Shanghai, China). DyLight 488-modified goat anti-mouse IgG was bought from Abbkine Scientific Co., Ltd (Georgia, USA). UltraSensitive^{TM} SP (Mouse/Rabbit) IHC Kit was bought from Fuzhou Maixin Biotech. Co., Ltd (Fuzhou, China). Chlorin e6 (Ce6) and methyl-β-cyclodextrin were bought from Shanghai yuanye Bio-Technology Co., Ltd (Shanghai, China). All the culture consumables needed for cell culture were bought from Gibco life technologies manufacturer (New York, USA). A Millipore Milli-Q purification system was utilized to produce deionized (DI) water (18.2 MΩ·cm) throughout the experiments.

Synthesis of 50 and 90 nm AuNPs: The AuNPs were prepared by a seed-mediated growth method. The first step is to prepare 13 nm Au seeds. In brief, 1 mL of $HAuCl_4$ aqueous solution (100 mM) and 100 mL of DI water were mixed in a three-necked round-bottomed flask and heated at 150 °C in an oil bath for 15 min under vigorous stirring. Then, 10 mL of sodium citrate aqueous solution (38.8 mM) was injected into the

above solution and kept it boiling for 10 min before cooling to room temperature. The second step is to grow the Au seeds. Briefly, 142.5 mL of DI water, 2 mL of sodium citrate aqueous solution (60 mM), and 7.5 mL of Au seeds (13 nm) were added into a three-necked round-bottomed flask (250 mL) and pre-heated at 90 ºC for 10 min under vigorous stirring. Then, 1 mL of $HAuCl_4$ aqueous solution (25 mM) was injected, and kept the mixture heated for 30 min. The process (addition of 1 mL of 25 mM $HAuCl_4$ aqueous solution) was repeated twice. By repeating the growth procedure described above, the 50 nm and 90 nm AuNPs can be obtained.

Synthesis of EAPNPs: The eccentric Au–PS nanoparticles (EAPNPs) were synthesized by using the method reported by Ohnuma. Briefly, 2.5 mL DI water, 1 mL of NaSS aqueous solution (6 mg/mL), 1 mL of KPS aqueous solution (50 mg/mL) and 16.5 mL of ethanol were mixed in a 25 mL three-necked flask. The mixture solution was preheated at 70 ºC in an oil bath under magnetic stirring for 10 min. Then, 0.2 mL of a mixture of styrene and DVB (200:1 v/v) was injected into the flask, and 2 min later, 3.0 mL of the 50 nm AuNPs solution was added. The reaction system was heated at 70 ºC for another 4 h. The resulting EAPNPs were washed several times with a mixture of DI water and ethanol and re-dispersed in 20 mL of DI water. Finally, the resulting EAPNPs were partially etched with a 10% v/v THF ethanol solution for 10 min to expose the surface of AuNPs.

Synthesis of biotin-modified Janus AuNPs: The biotin-modified Janus AuNPs were synthesized using a stepwise coupling method according to our previous work[1]. In brief, 1 mL of HS-PEG-Biotin aqueous solution was injected into 2 mL of the partially etched EAPNPs aqueous solution and the mixture was stirred at room temperature for 12 h. The products were washed with ethanol for several times, followed by dissolving in pure THF to remove the PS hemispheres. The resulting product was washed with DI water several times and re-dispersed in 2 mL of DI water. Finally, 1 mL of HS-PEG-OCH_3 aqueous solution was injected into the above solution and the mixture was stirred at room temperature for 12 h. The as-obtained biotin-modified Janus AuNPs were washed with DI water for several times and re-dispersed in 1 mL of DI water for further use.

Synthesis of biotinylated enzyme molecules: Biotinylated enzyme molecules were prepared by the amide-coupling reaction following the previously reported protocol[2]. In brief, 100 μL of urease, catalase, or glucose oxidase and 400 μL of N-Succinimidyl-6-Biotinamidohexanoate were mixed and incubated at room temperature under stirring for 24 h. The as-obtained biotinylated enzyme molecules were washed several times with PB buffer solution, re-dispersed in 100 μL PB buffer solution, and stored at 4 °C for further use.

Preparation of CUPJNRs: The biotinylated enzyme molecules were sequentially assembled onto the

surface of the biotin-modified Janus AuNPs using the streptavidin as cross-linkers. In brief, 1 mL streptavidin was added into 1 mL of biotin-modified Janus AuNPs and the mixture solution was incubated at room temperature under stirring for 12 h. The products were washed several times with DI water and re-dispersed in 1 mL of PBS buffer solution. Then, 1 mL of biotinylated catalase was added into the above solution and the mixture solution was incubated at room temperature under stirring for 12 h. The intermediate products were washed several times with DI water, and re-dispersed in 1 mL of PBS buffer solution. Then, by repeating the above process with biotinylated urease, the resulting CUPJNRs were obtained after they were washed several times with DI water, re-dispersed in 1 mL of DI water, and stored at 4 ºC for further characterization. The C*UPJNRs, CU*PJNRs and C*U*PJNRs were prepared by using the inactive ureases and catalases following the same method. The inactive ureases were prepared by heating the urease solution in a 95°C water bath for 30 min. The inactive catalases were prepared by heating the urease solution in an 80°C water bath for 30 min. The SA-QDs-modified CUPJNRs were prepared by using the SA-QDs following the same method.

Motion behavior of CUPJNRs: In brief, 1 μL of CUPJNRs aqueous suspension was added onto an ultrasonically cleaned cover glass, followed by dropping 10 μL of fuel solution (dissolved in different media). An inverted dark-field microscopy (Zeiss HAL100) with 50× objective magnification at a frame rate of about 18 fps was utilized to observe and record the motion behavior of CUPJNRs. A commercial software (Video Spot Tracker) was applied to record the movement trajectories and the corresponding coordinates of CUPJNRs. Thereafter, MSD was calculated using the following formula: $MSD(\Delta t) = [(x_i(t + \Delta t) - x_i(t))^2]$ ($i = 2$, for two-dimensional analysis). Subsequently, the formula ($MSD(\Delta t) = 4D_{eff}\Delta t$) was used to obtain the effective diffusion coefficient (D_{eff}) of the nanorobots.

***In vitro* tumor-targeting behavior of CUPJNRs:** A microfluidic chip (from FluidicLab) was used to evaluate the *in-vitro* tumor-targeting behavior of CUPJNRs in a 3D cell model. The left reservoir (zone 1) was filled with 40 μL of gel matrix containing MCF-7 cells (tumor cells), while the right reservoir (zone 2) was filled with 40 μL of gel matrix containing MCF-10A cells (normal cells). 40 μL of the mixture solution was added into the reservoirs of the microfluidic chip and solidified to form collagen gels that contained cells. After that, 100 μL of mouse plasma including 10 mM urea was added into the straight channel and 20 μL of nanorobots were injected in the middle reservoir. The H_2O_2 diffusion in the straight channel was monitored with a H_2O_2 fluorescence probe (Bestbio). The collective behavior of the nanorobots was measured with a microplate reader in zones 3, 4, and 5, while the individual behavior of the nanorobots was observed with an inverted dark-field microscope in zones 4 and 5.

Quantification of urease activity: The enzyme activity of the urease molecules immobilized on the surface of the CUPJNRs was quantified using an established method[3]. The method is based on the detection of ammonia produced by the catalytic decomposition of urea by urease, which induces a color change of the reaction solution from yellow to blue/green. In brief, 10 μL of CUPJNRs (0.1 nM) aqueous suspension was added into 90 μL of urea aqueous solution (20 mM) or a mixed solution of urea (20 mM) and H_2O_2 (200 mM) and the mixture was incubated at 25 °C for 30 min. Then, the ammonia concentration in the reaction solution was measured following the protocol in reference.

Quantification of catalase activity: The enzyme activity of the catalase molecules immobilized on the surface of the CUPJNRs was quantified using a commercial catalase activity assay kit (Elabscience)[4]. In brief, 10 μL of CUPJNRs (0.1 nM) aqueous suspension was added into 90 μL of H_2O_2 aqueous solution (200 mM) or a mixed solution of urea (20 mM) and H_2O_2 (200 mM) and the mixture was incubated at 25 °C for 30 min. Then, a catalase activity assay kit was used to detect the content of H_2O_2 in it.

Chemotaxis of CUPJNRs: A μ–slide microfluidic chip (from Ibidi) was used to evaluate the chemotaxis of CUPJNRs. A piece of agarose gel containing H_2O_2 aqueous solution (1 M) was added in the left reservoir to establish a H_2O_2 concentration gradient along the channel, while the straight channel was filled with a urea aqueous solution with different concentrations. CUPJNRs were injected into the right reservoir. The collective chemotactic behavior of CUPJNRs was measured with a microplate reader in zones 2 and 3, while the individual chemotactic behavior of CUPJNRs was observed with an inverted dark-field microscope in zone 4. The absorbance at 530 nm (for 50 nm nanorobots) and 560 nm (for 90 nm nanorobots) were measured to quantify the concentration of CUPJNRs in different zones of the μ–slide microfluidic chip. The motion trajectory and corresponding coordinates of the nanorobots were recorded using the Video Spot Tracker software. These coordinates were then input into the Chemotaxis and Migration Tool 2.0 software to calculate the directionality and DCM.

A micropipette (from Gairdner SCIENTIFIC) with an inside diameter of 18 μm was used to evaluate the chemotactic sensitivity of CUPJNRs. The micropipette filled with a H_2O_2 solution was fixed on the holder of a high-precision micromanipulator (Leica Microsystems, Germany) and then was inserted into a CUPJNRs-containing droplet (~120 μL) on the cover glass. The other side of the micropipette was connected to a highly airtight oil pump to prevent the leakage of solution. The chemotactic behavior of CUPJNRs inside the dashed box was recorded by an inverted dark-field microscope. The ratio of nanorobots within the dashed boxes in Fig. 3h under different conditions was calculated using the following formula:

$$\text{Ratio of nanorobots} = \Delta N_{urea\,(+)}/\Delta N_{urea\,(-)}$$

Here, $\Delta N_{urea\,(+)}$ is the increased number of nanorobots within the dashed boxes (with 20 mM urea), and $\Delta N_{urea\,(-)}$ is the increased number of nanorobots within the dashed boxes (without urea). The increased number of nanorobots within the dashed box after different treatments was shown in **Figs. S25–27**.

Dynamic chemotactic behavior of CUPJNRs: To evaluate the *in-vitro* dynamic chemotactic behavior of CUPJNRs in a 3D cell model, a microfluidic chip was purchased from Ibidi. The two reservoirs are respectively located at the two sides of the channel, and connected to the channel through small holes in a distance of 6.5 mm. One reservoir was filled with 100 µL of gel matrix containing MCF-7 cells (tumor cells), while the other was filled with 100 µL of gel matrix containing MCF-10A cells (normal cells). After that, a mouse plasma which contains 90 nm Cy5-modified nanorobots with different formulations was injected into the middle channel via a pump. The flow velocity of the mouse plasma in the channel was controlled as 0.2 mL/h. The fluorescence images of zones 5 (tumor cell zone) and 6 (normal cell zone) were recorded using a Zeiss confocal fluorescence microscopy (Zeiss LSM 900, Germany) at ×20 magnification. Quantitative fluorescence analysis of images was measured using *Image J*. The volume flow rate in dynamic microfluidic model using the below equation.

$$V_{LVOT} = \mathrm{Q}/CSA_{LVOT}$$

where V_{LVOT} is the outflow tract velocity of a mass point in the blood vessel, and Q is the volume flow rate to circular crosssectional area (CSA_{LVOT}) of a single-strand channel. The flow velocity of blood within the capillaries is ranged from 300 to 700 µm/s. When the volume flow rate Q was 0.2 mL/h, the flow velocity of fluid within the channel was about 300 µm/s.

Numerical simulation: To investigate the concentration distribution of H_2O_2 molecules in a μ-slide microfluidic channel or around a micropipette, we simulated the time-dependent diffusion of H_2O_2 molecules in a two-dimensional plane following equation (1)[5].

$$\partial t\, \phi(x,\ y,\ t) = D\, (\partial 2x + \partial 2x)\, \phi(x,\ y,\ t) + c\, \phi(x,\ y,\ t) \qquad (1)$$

Here $\phi(x, y, t)$ denotes the concentration distribution of H_2O_2 molecules in the *xy* plane at time *t*. *D* is the diffusion coefficient of H_2O_2 molecules, which is set as a constant during simulation. The simulation was performed by COMSOL Multiphysics software (version 6.0).

When CUPJNRs were immersed in H_2O_2 aqueous solutions, the catalase on the surface of the CUPJNRs decomposed H_2O_2 into O_2 and H_2O. The distribution of O_2 molecules originates from its flux (J_i) from the surface of CUPJNRs and is further influenced by convection and migration (equation (2)). In our model, this was solved with the conservation equation (equation (3)) at a steady state.

$$J_i = uc_i - D_i \nabla c_i - \frac{z_i F D_i c_i \nabla \varphi}{RT} \qquad (2)$$

$$\nabla \cdot J_i = 0 = u \cdot \nabla c_i - D_i \nabla^2 c_i - \frac{z_i F D_i \nabla \cdot (c_i \nabla \varphi)}{RT} \quad (3)$$

where u is the fluid velocity, F is the Faraday constant, φ is the electrostatic potential, R is the gas constant, T is the absolute temperature, and c_i, D_i, z_i are the concentration, diffusion coefficient, and charge of species i, respectively.

The inertial effect is neglected in the present study because of a very small Reynolds number. Thus, the flow field is governed by the Stokes equations,

$$-\nabla p + \mu \nabla^2 u = 0 \quad (4)$$

and the continuity equation for the incompressible fluid,

$$\nabla \cdot u = 0 \quad (5)$$

In these equations, u is the fluid velocity vector, and p is the pressure. The initial values of the flow velocity and the pressure are all zero.

To calculate the M_p on the CUPJNR, the forces of the flow acting on the surface of the CUPJNRs (F_x and F_y) were at first decomposed into a force tangential to the motor surface (F_t) and that along the radius direction (F_p),

$$F_t = \frac{y}{r} F_x + \frac{x}{r} F_y \quad (6)$$

$$F_p = \frac{y}{r} F_y + \frac{x}{r} F_x \quad (7)$$

Then, M_p was calculated as follows,

$$M_p = F_t r \quad (8)$$

By calculating the M_p and F_p at different fuel concentrations and comparing them with Brownian force, the motion behaviors of nanorobots under different conditions are derived.

Preparation of the nanorobots with catalases and ureases asymmetrically modified on the two opposite sides of AuNPs: The nanorobots with catalases and ureases asymmetrically modified on the two opposite sides of AuNPs were synthesized using a stepwise coupling method according to our previous work[1]. In brief, the PEG(COOH)-modified Au-PS eccentric nanoparticles were washed with ethanol for several times, followed by dissolving in pure THF to remove the PS hemispheres. The resulting product was washed with DI water several times and re-dispersed in 2 mL of DI water. 1 mL of HS-PEG-Biotin aqueous solution was injected into the above solution and the mixture was stirred at room temperature for 12 h.

Then, 20 µL of 80 mM EDC solution and 40 µL of 80 mM NHS were introduced into the above solution and shaken at 25 °C. After 30 min, 100 µL of aqueous catalase solution was added and incubated overnight.

The intermediate products were washed several times with DI water, and re-dispersed in 1 mL of PBS buffer solution.

Finally, 1 mL streptavidin (SA) was added into the above solution and the mixture solution was incubated at room temperature under stirring for 12 h. The products were washed several times with DI water and re-dispersed in 1 mL of PBS buffer solution. Then, 1 mL of biotinylated urease was added into the above solution and the mixture solution was incubated at room temperature under stirring for 2 h. The resulting the nanorobots with catalases and ureases asymmetrically modified on the two opposite sides of AuNPs were obtained after they were washed several times with DI water, re-dispersed in 1 mL of DI water, and stored at 4 °C for further characterization.

Chemotaxis of the nanorobots with catalases and ureases asymmetrically modified on the two opposite sides of AuNPs: A μ–slide microfluidic chip was used to evaluate the chemotaxis of the nanorobots with catalases and ureases asymmetrically modified on the two opposite sides of AuNPs (**Figs. S30**). A piece of agarose gel containing H_2O_2 aqueous solution (1 M) was added in the left reservoir to establish a H_2O_2 concentration gradient along the channel, while the straight channel was filled with a 0 or 20 mM urea aqueous solution. The individual chemotactic behavior of the nanorobots with catalases and ureases asymmetrically modified on the two opposite sides of AuNPs was observed with an inverted dark-field microscope in zone 4. The motion trajectory and corresponding coordinates of the nanorobots were recorded using the Video Spot Tracker software.

***In vivo* biodistribution measurement of CUPJNRs:** A tumor-bearing mouse model was established with C57BL/6 mice (male, 6−8 weeks old) to evaluate the *in vivo* tumor-targeting capability of CUPJNRs after intravenous injection. Briefly, 150 μL of B16F10 cells (2.5×10^8 cells mL^{-1}) were subcutaneously injected into the back of mice. When the volume of tumor reached about 300 mm^3, the mice were randomly divided into four groups (n = 4), and intravenously injected with different samples (0.5 mg/kg in 200 μL PBS), or 200 μL PBS (the control). At the endpoint of the experiments, the mice were euthanized, and the tumors, blood, and major organs (heart, liver, spleen, lung and kidney) were separately collected, and then disrupted with aqua regia for measuring the Au content by ICP−MS. The biodistributions of the injected samples were calculated by the percentage of the injected dose per gram of tissue (% ID/g). All animal experiments were approved by the institutional Animal Care and Use Committee at Wuhan University of Technology (Reg. No. WHUT2022-008) and performed following European Community Guidelines (2010/63/EU). The delivery efficiency was calculated using the following formula:

$$\text{Delivery efficiency (\%ID)} = (W_N/ID)*100$$

$$\text{Delivery efficiency (\%ID/g)} = (W_N/ID)*100/W_T$$

Here, W_N is the weight of nanorobot within tissue, W_T is the weight of tissue, and ID is the injection dosage of nanorobot.

Detection of H_2O_2 content in tissue: The collected tumor tissues and lungs were embedded in OCT mounting medium, placed in a cryostat machine and dissected into 7-μm sections. Sections were stained with H_2O_2 staining solution (BIOESN, BES20249BO) and DAPI solution. Subsequently, the sections were scanned with a tissue scanner (3DHISTECH CaseViewer, Pannoramic SCAN) and processed with Pannoramic viewer software. ImageJ software was used to analyze the fluorescence intensity.

Synthesis of Cy5-modified CUPJNRs (Cy5-CUPJNRs) and FITC-modified CUPJNRs (FITC-CUPJNRs): Cy5 was modified onto the surface of CUPJNRs by an amide reaction. In briefly, 1 mL of Cy5-NHS ester aqueous solution was added into 1 mL of CUPJNRs suspension and shaken at 25 °C for 12 h. The as-obtained products were washed several times with DI water, and stored in DI water at 4 °C for further usage. The FITC-CUPJNRs were prepared by using the 5/6FAM-NHS following the same method.

Penetration capability of CUPJNRs: A tumor-bearing mouse model was established with BALB/c mice (female, 4–6 weeks old) to evaluate the penetration capability of nanorobots *in vivo*. Briefly, 0.1 mL of 4T1 cells (2×10^7 cells/mL) were subcutaneously injected into the back of mice. When the volume of tumor reached about 100 mm^3, the mice were randomly divided into five groups (n = 6), and injected from tail vein with 200 μL of different samples (G1: PBS; G2: C*U*PJNRs; G3: C*UPJNRs; G4: CU*PJNRs; and G5: CUPJNRs). The nanorobots with different formulations were modified with Cy5. The tumors were excised and sectioned with a thickness of 4 μm. The UltraSensitive™ SP (Mouse/Rabbit) IHC Kit was used to block non-specific antigen binding on the tissue surface. Then the primary antibody (CD34 monoclonal antibody (RAM34)) was added dropwise and incubated overnight at 4 °C to target the blood vessels. After washing, the secondary antibody (DyLight 488, Goat Anti-Mouse IgG) was added and incubated for 2 hours to label CD34 monoclonal antibody. The nucleic were stained with Hoechst 33342. The fluorescence images were recorded using a Zeiss confocal fluorescence microscopy (Zeiss LSM 900, Germany) at ×40 magnification. Quantitative fluorescence analysis of images were measured using *Image J.*

Intratumoral penetration capability of CUPJNRs: A tumor-bearing mouse model was established with BALB/c mice (female, 4–6 weeks old) to evaluate the penetration capability of nanorobots *in vivo*. Briefly, 0.1 mL of 4T1 cells (2×10^7 cells/mL) were subcutaneously injected into the back of mice. When the volume of tumor reached about 200 mm^3, the mice were randomly divided into two groups (n = 2), and intratumorally injected with 200 μL of different samples (the mixture of Cy5-C*UPJNRs and FITC-C*U*PJNRs or the

mixture of Cy5-CU*PJNRs and FITC-C*U*PJNRs). At 24 h post administration, the tumors were excised and sectioned into 10 μm slices. The nuclei were stained with DAPI. The fluorescence images were recorded using a Zeiss confocal fluorescence microscopy (Zeiss LSM 900, Germany) at ×20 magnification. Quantitative fluorescence analysis of images were measured using *Image J*.

Internalization of CUPJNRs by tumor-associated cells and flow cytometry: A tumor-bearing mouse model was established with BALB/c mice (female, 4–6 weeks old) to evaluate the penetration capability of nanorobots in vivo. Briefly, 0.1 mL of 4T1 cells (2×10^7 cells/mL) were subcutaneously injected into the back of mice. When the volume of tumor reached about 100 mm^3, the mice were randomly divided into five groups (n = 6), and injected from tail vein with 200 μL of different samples (G1: PBS; G2: C*U*PJNRs; G3: C*UPJNRs; G4: CU*PJNRs; and G5: CUPJNRs). The nanorobots with different formulations were modified with Cy5. Tumors were manually diced with a scissor into ≤1mm^3 pieces and then transferred into 5 mL of a digestion solution containing PBS with 400 μg/ml collagenase IV and incubated for 60 min under gentle rotation at 37 °C. Disaggregated cells were filtered through a 70 μm mesh strainer, then centrifuged at 500 g for 5 min. The cells were washed 2 times at 500 g for 5 min using PBS buffer solution supplemented. Live/dead staining was performed with live/dead fixable aqua dead cell stain kit according to the manufacturer's instructions. The immune cells were labelled with APC/Cyanine7 anti-mouse CD45 antibody. The cells were washed 2 times at 500 g for 5 min using PBS buffer solution. Cells were fixed with 4% paraformaldehyde in PBS buffer solution for 30 min, then washed once and stored at 4 °C before flow cytometry analysis. Events were acquired with a CytoFLEX Flow Cytometer (Beckman, California, US). Data were analysed using FlowJo 10.8.1 (TreeStar Inc.). The enhanced rate of the percentage of nanorobot-positive CD45-negative tumor-associated cells was calculated using the following formula:

$$ER_p = (P_{CUPJNR} - P_{PBS})/(P_{control} - P_{PBS}).$$

Here, P_{CUPJNR} represents the percentage of CUPJNR-positive CD45-negative tumor-associated cells, $P_{control}$ denotes the percentage of control group-positive CD45-negative tumor-associated cells, and P_{PBS} is the background signal. The enhanced rate of nanorobots uptake within CD45-negative tumor-associated cells was determined using the following formula:

$$ER_{MFI} = (MFI_{CUPJNR} - MFI_{PBS})/(MFI_{control} - MFI_{PBS}).$$

Here, MFI_{CUPJNR} is the average FI of Cy5 in the CUPJNR-treated group, $MFI_{control}$ represents the average FI of Cy5 in the control group, and MFI_{PBS} signifies the average FI of Cy5 in the PBS-treated group.

Cellular uptake of the CUPJNRs: MCF-7 cells were seeded at a density of 8000 cells per 10 mm confocal dish and incubated at 37 ºC for 24 h. The old medium was then replaced with 300 μL of methyl-β-

cyclodextrin (5 mM), cytochalasin D (5 μM) or hypertonic sucrose (0.45 M) dissolved in DMEM. Additionlly, one confocal dish was kept as 4°C. After incubation for 30 min, the medium was added with 300 μL of Cy5-CUPJNRs or Cy5-C*U*PJNRs (50 μg/mL) suspension (dissolved in DMEM with 20 mM urea). After further incubation for 3 h, the cells were stained with the Hoechst33342 at 37 °C for 30 min. Finally, The cells were washed with PBS buffer solution and observed by Zeiss confocal fluorescence microscopy (Zeiss LSM 900, Germany). Quantitative fluorescence analysis of images were measured using *Image-Pro Plus*.

Preparation of Ce6-loaded CUPJNRs (Ce6-CUPJNRs): Ce6-CUPJNRs were prepared by conjugating Ce6 to CUPJNRs through an amide reaction. In brief, 120 μL of Ce6, 10 μL of EDC, and 20 μL of NHS were mixed and incubated at room temperature for 30 min, and then added into 1 mL of 0.01 nM CUPJNRs. The mixture solution was incubated at room temperature overnight to get the resultant product Ce6-CUPJNRs, which were washed several times with DI water, re-dispersed in 1 mL of PBS buffer solution, and stored at 4 °C for further use.

***In vivo* antitumor efficacy of Ce6-CUPJNRs**: A tumor-bearing mouse model was established with BALB/c mice (female, 4−6 weeks old) to evaluate the antitumor efficacy of nanorobots *in vivo*. Briefly, 0.1 mL of 4T1 cells (2×10^7 cells/mL) were subcutaneously injected into the back of mice. When the tumor volume reached about 100 mm^3, the mice were randomly divided into five groups (n = 6), and injected from tail vein with 200 μL of different samples (G1: PBS; G2: Ce6-C*U*PJNRs; G3: Ce6-C*UPJNRs; G4: Ce6-CU*PJNRs; and G5: Ce6-CUPJNRs) every 3 days for a total of 3 doses. Each mice was injected with 3 μg/kg of Ce6. After 24 h post intravenous injection, the tumors were exposed to a 660 nm laser irradiation at a power density of 0.18 W/cm^2 for 10 min. The tumor volume and weight of mice were monitored during the treatment course. The mice were euthanized at the end point of treatment (day 18). The gauged volume (mm^3) was calculated with $0.5 \times \text{length} \times \text{width}^2$. TGI rate was calculated as TGI% = $(1-V_T/V_C) \times 100\%$, where V_T and V_C were the mean tumor volume of the treated and control groups, respectively. The typical tumors of different groups were embedded and sectioned in paraffin wax, and then conducted H&E, TUNEL and Ki67 staining. The blood biochemical examination was evaluated by an automatic biochemical analyzer (MNCHIP, Pointcare V3). H&E staining of the main organs was performed for the histological analysis.

***In vivo* antitumor efficacy of Ce6**: A tumor-bearing mouse model was established with BALB/c mice (female, 4−6 weeks old) to evaluate the antitumor efficacy of nanorobots *in vivo*. Briefly, 0.1 mL of 4T1 cells (2×10^7 cells/mL) were subcutaneously injected into the back of mice. When the tumor volume reached about 100 mm^3, the mice were randomly divided into five groups (n = 6), and injected from tail vein with 200 μL of different samples (G1: PBS; G2: Ce6; G3: CUPJNRs) every 3 days for a total of 3 doses. Each mouse in the

G2 was injected with 5 mg/kg of Ce6 and each mouse in the G3 was injected with 0.5 mg/kg of CUPJNR. After 24 h post intravenous injection, the tumors were exposed to a 660 nm laser irradiation at a power density of 0.18 W/cm^2 for 10 min. The tumor volume and weight of mice were monitored during the treatment course. The mice were euthanized at the end point of treatment (day 18). The gauged volume (mm^3) was calculated with 0.5 × length × $width^2$. TGI rate was calculated as TGI% = $(1-V_T/V_C) \times 100\%$, where V_T and V_C were the mean tumor volume of the treated and control groups, respectively.

Instruments: TEM images were taken by a JEOL transmission electron microscope (JEOL JEM-F200, Japan). SEM images were captured by a Hitachi scanning electron microscope (Hitachi S-4800, Japan). A Zeiss inverted dark-field microscopy (Zeiss HAL100, Germany) was applied to record the motion behaviors of nanorobots at room temperature. A UV-2550 spectrometer (Shimadzu, Japan) was used to obtain the UV-Vis absorption spectra of the samples. The absorbance of the samples was measured by a microplate reader (Perkin Elmer EnSight HH3400, USA) in the CCK-8 assay. A Malvern Zetasizer instrument (Zetasizer Nano ZS90, England) was used to measure the hydrodynamic size and zeta potential of the samples. The concentration of nanorobots was measured by ICP−MS (PerkinElmer NexION 2000, USA). The H_2O_2 content in tissues was measured by Pannoramic SCAN (3DHISTECH CaseViewer, Hungary).

Statistical analysis: Data were expressed as mean ± standard deviation (SD) or mean ± standard error of the mean (SE). Differences between groups were analyzed using one-way analysis of variance (ANOVA) followed by Tukey's test using the Prism 9.0 software (GraphPad Inc., San Diego CA, USA). Statistical significance was indicated as ****P < 0.0001, **P < 0.05 and n.s., not significant.

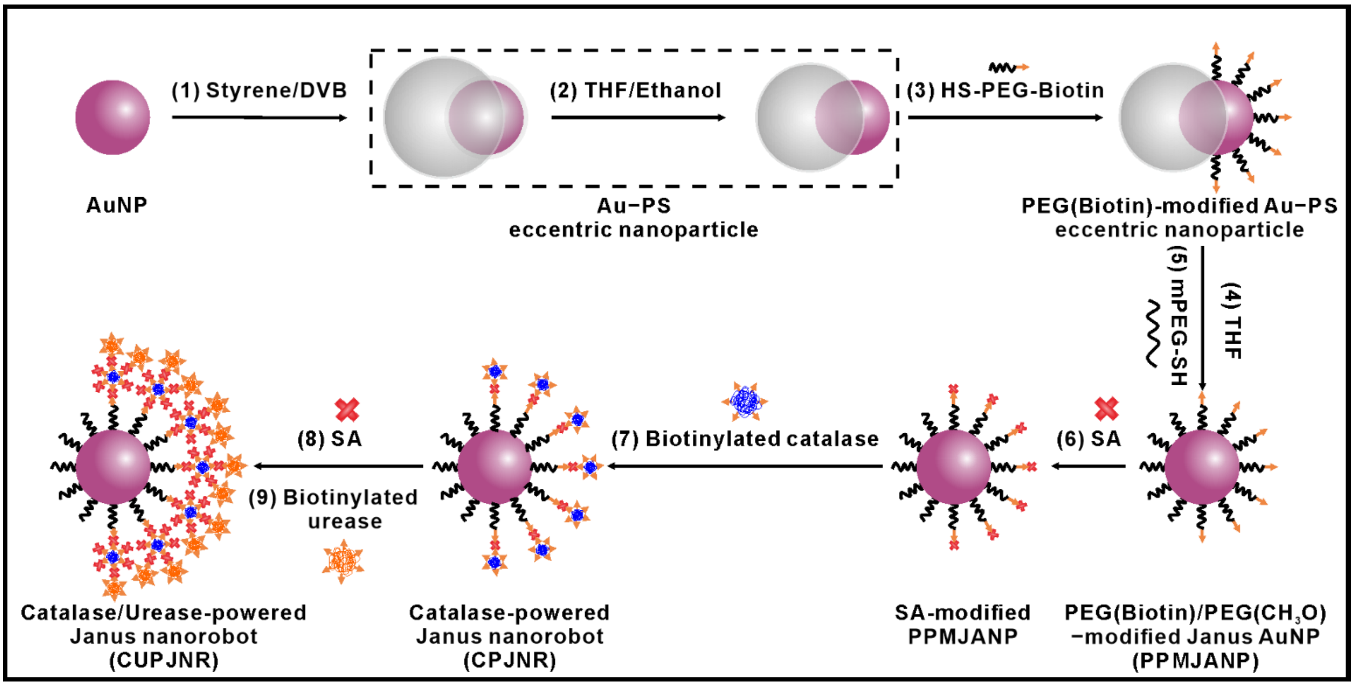

Supplementary Figure 1. Schematic illustration of the preparation procedure used to fabricate the CUPJNRs.

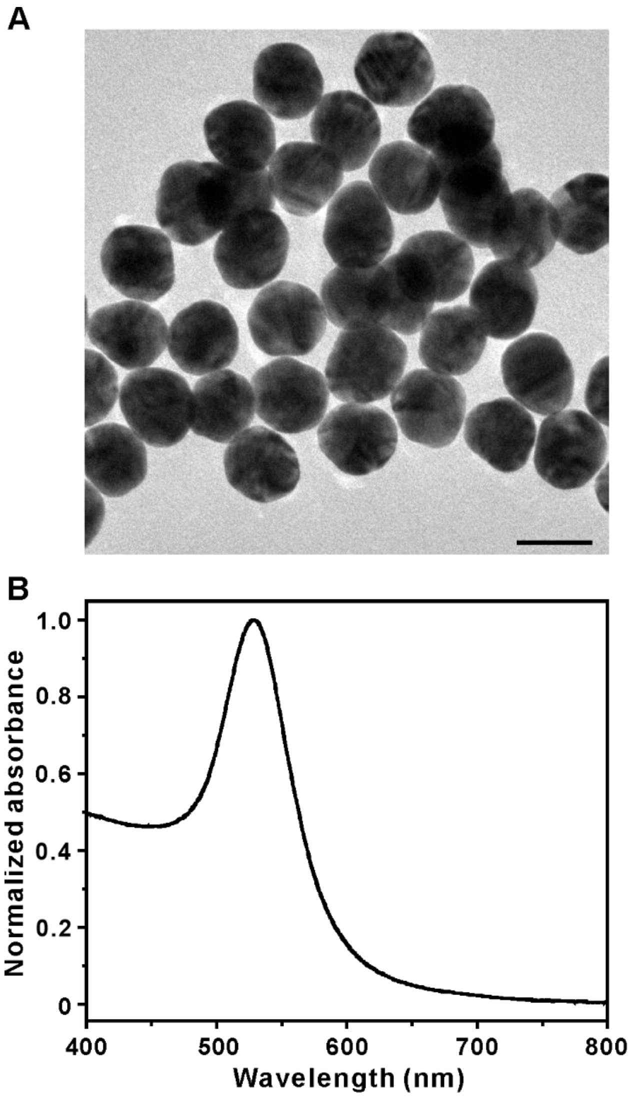

Supplementary Figure 2. (A) TEM images and (B) UV-Vis absorption spectrum of 50 nm AuNPs. Scale bar: 50 nm.

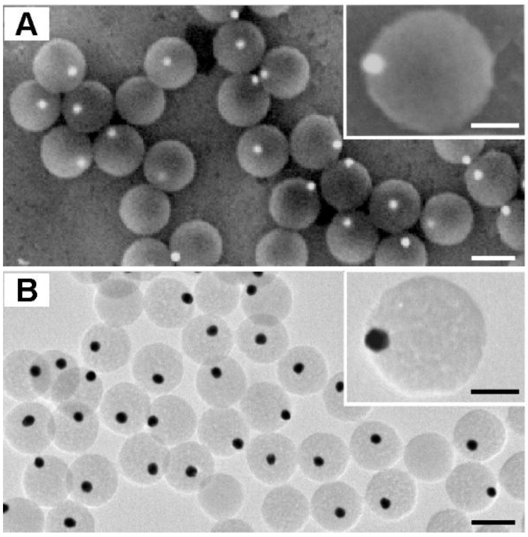

Supplementary Figure 3. (A) SEM and (B) TEM images of the eccentric Au–PS nanoparticles (EAPNPs) prepared by 50 nm AuNPs. Scale bars: 500 nm, 100 nm (inset).

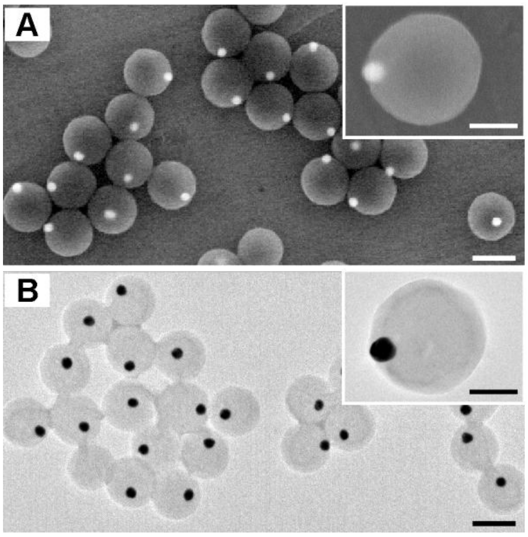

Supplementary Figure 4. (A) SEM and (B) TEM images of the partially etched EAPNPs by THF. Scale bars: 500 nm, 100 nm (inset).

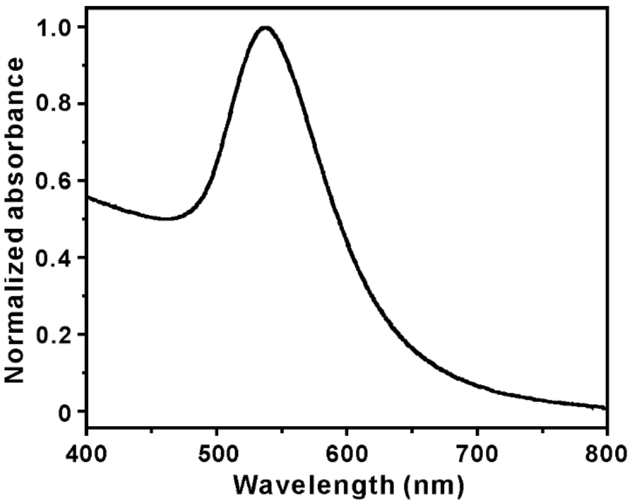

Supplementary Figure 5. UV-Vis absorption spectrum of 50 nm CUPJNRs.

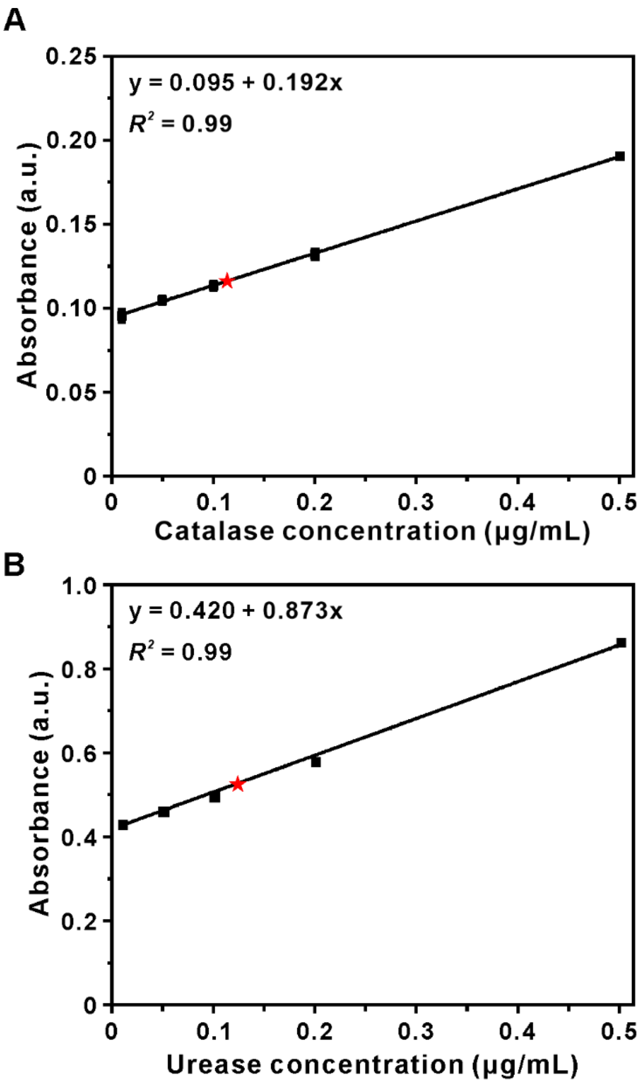

Supplementary Figure 6. (A) The absorbance at 405 nm of detection solution *versus* the concentration of catalase [n = 3; mean ± SD]. (B) The absorbance at 687 nm of detection solution *versus* the concentration of urease [n = 3; mean ± SD]. Red asterisks represent the average absorbance of detection solution after addition of 0.05 nM 50 nm CUPJNRs [n = 3].

Discussion:

The number of catalases and ureases on the surface of CUPJNR using the below equation.

$$N_{enzyme} = (C_{enzyme} * V_{solution})/(M_{enzyme} * N_{CUPJNR})$$

where N_{enzyme} is the number of enzyme on each CUPJNR, C_{enzyme} is the concentration of enzymes on the surface of CUPJNRs in the test solution ($C_{catalase}$ is 0.11 μg/mL and C_{urease} is 0.12 μg/mL), $V_{solution}$ is the volume of the test solution ($V_{solution}$ is 1 mL), M_{Enzyme} is the relative molecular mass of the enzyme ($M_{catalase}$ is about 240000 and M_{urease} is about 480000), and N_{CUPJNR} is the number of CUPJNRs in the test solution (N_{CUPJNR} is 0.05 nM). The number of catalases and ureases on CUPJNR was about 9 and 5.

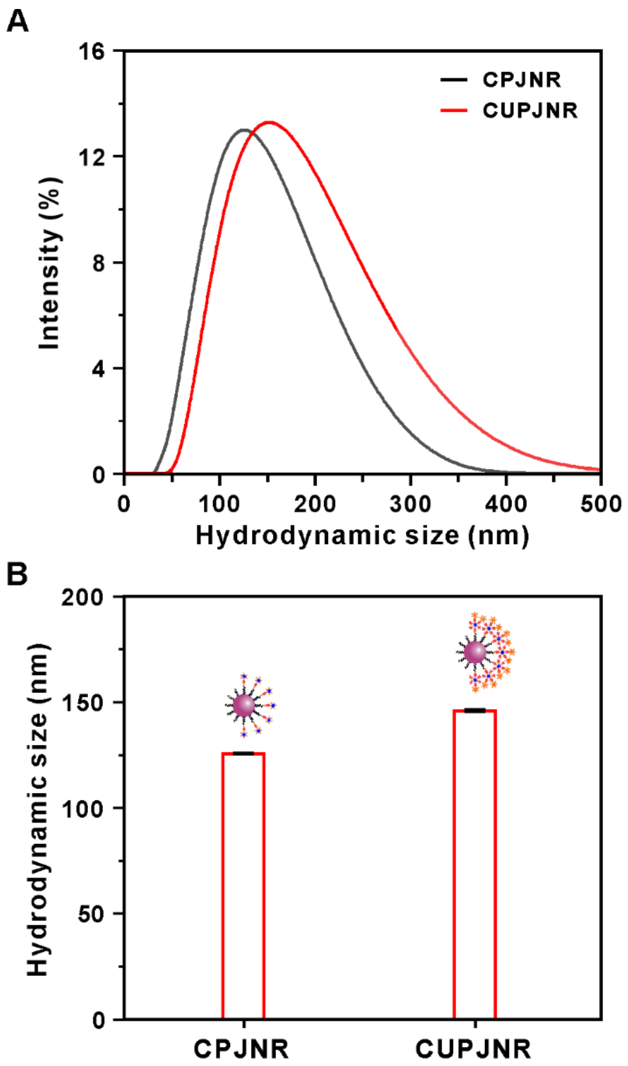

Supplementary Figure 7. (A) Hydrodynamic size distribution [n = 3] and (B) hydrodynamic size of the products during the preparation of CUPJNRs [n = 3; mean ± SD].

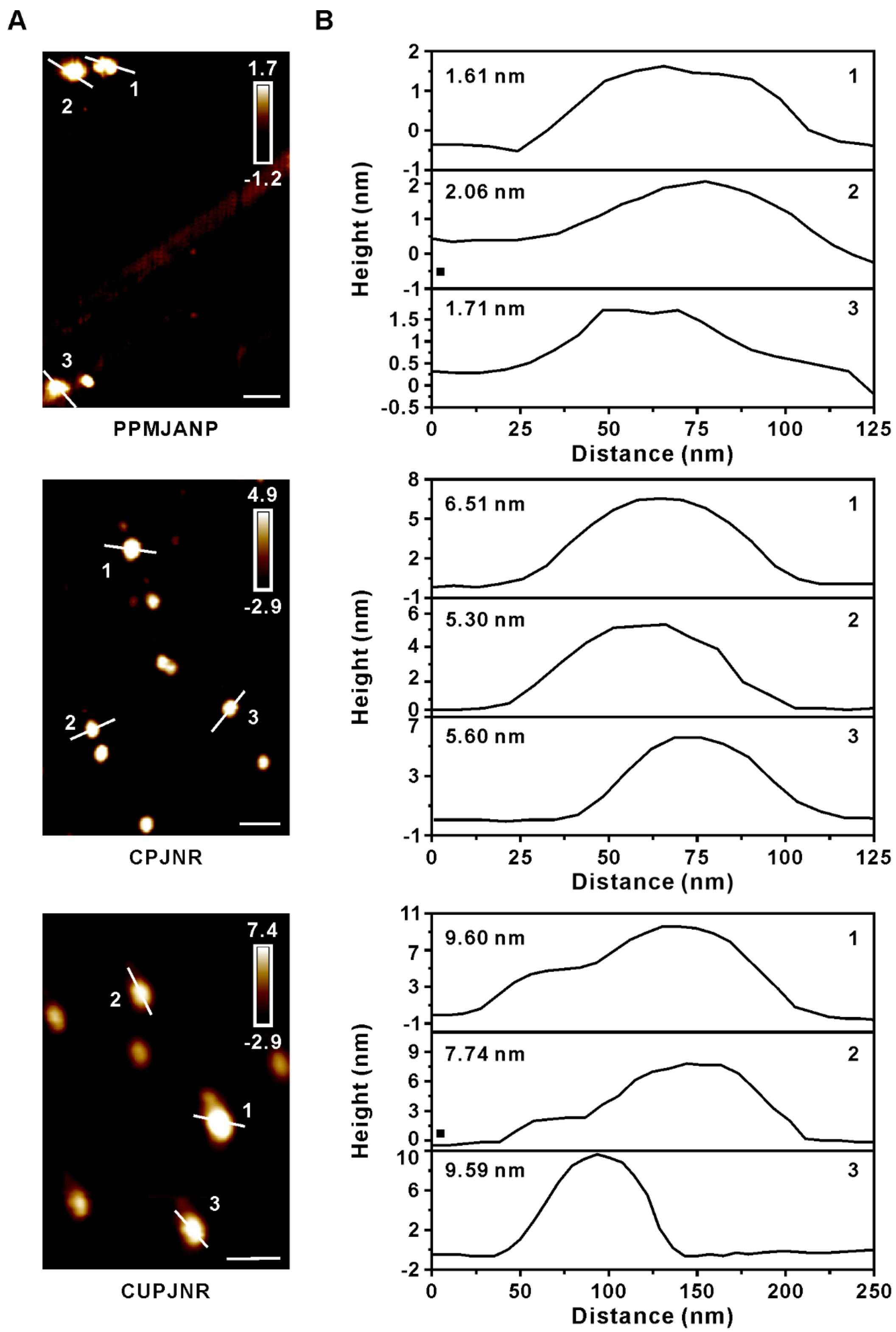

Supplementary Figure 8. (A) The 2D morphology images and (B) height profile distribution of the products during the preparation of CUPJNRs.

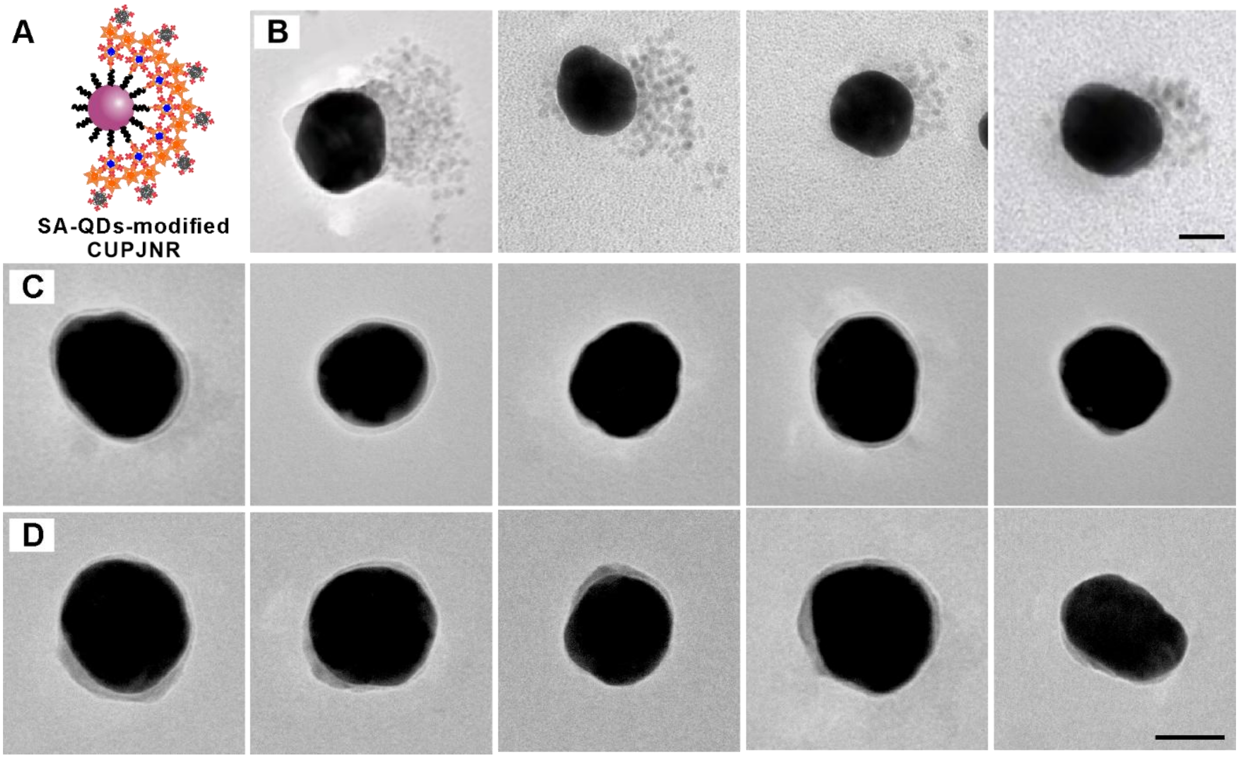

Supplementary Figure 9. (A) Schematic illustration and (B) high-magnification TEM image of SA-QDs asymmetrically assembled CUPJNRs. High-magnification TEM images of c, PEG-modified AuNP and d, CUPJNR. Scale bar: 50 nm.

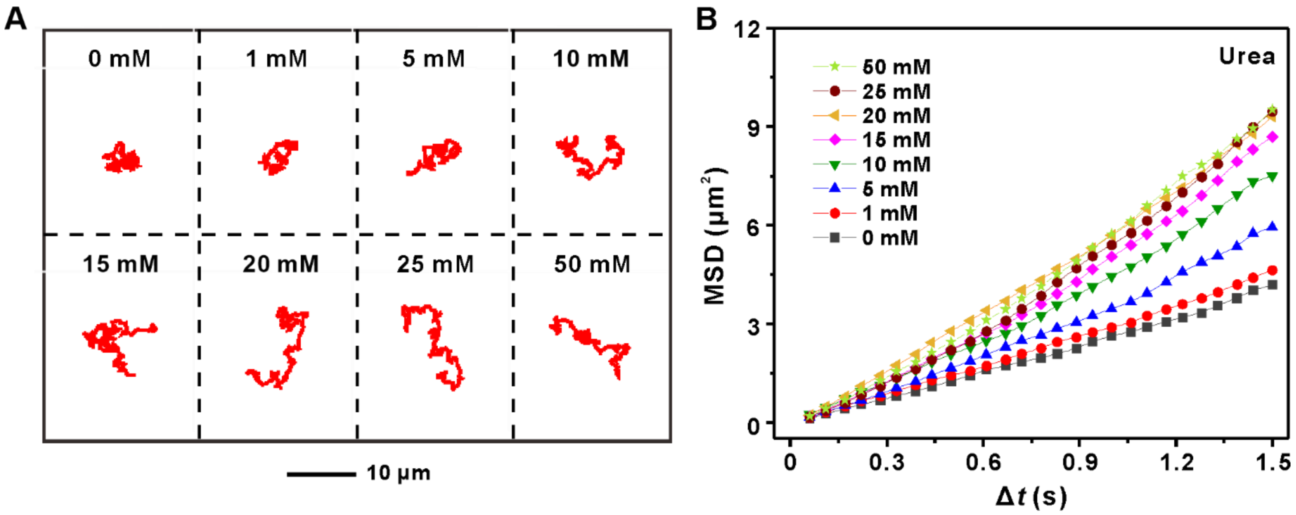

Supplementary Figure 10. Motion behavior of the 50 nm CUPJNRs in urea aqueous solution. (A) Typical trajectories of the CUPJNRs at different urea concentrations over a 10 s period, (B) corresponding mean-square-displacement (MSD) of the CUPJNRs *versus* the time interval (Δ*t*). The trajectories are obtained from Supplementary Video 1.

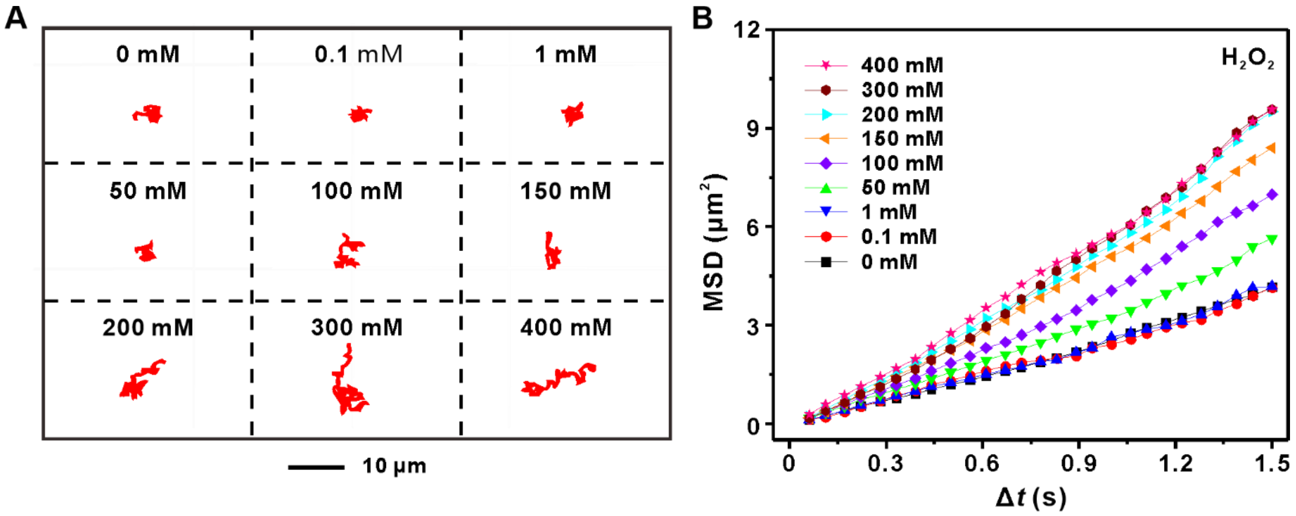

Supplementary Figure 11. Motion behavior of the 50 nm CUPJNRs in H_2O_2 aqueous solution. (A) Typical trajectories of the CUPJNRs at different H_2O_2 concentrations over a 10 s period. (B) Plots of MSD *versus* Δ*t*. The trajectories are obtained from Supplementary Video 2.

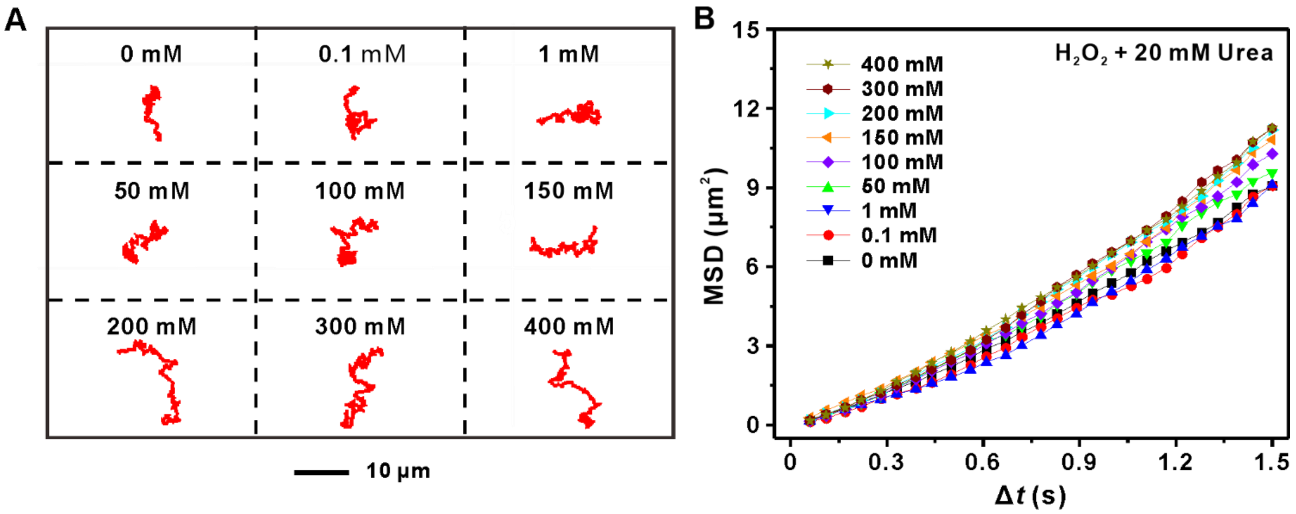

Supplementary Figure 12. Motion behavior of the 50 nm CUPJNRs in the mixed solution of urea and H_2O_2. (A) Typical trajectories at different H_2O_2 concentrations over a 10 s period. The urea concentration in the mixed solutions is 20 mM. (B) Plots of MSD *versus* Δt. The trajectories are obtained from Supplementary Video 3.

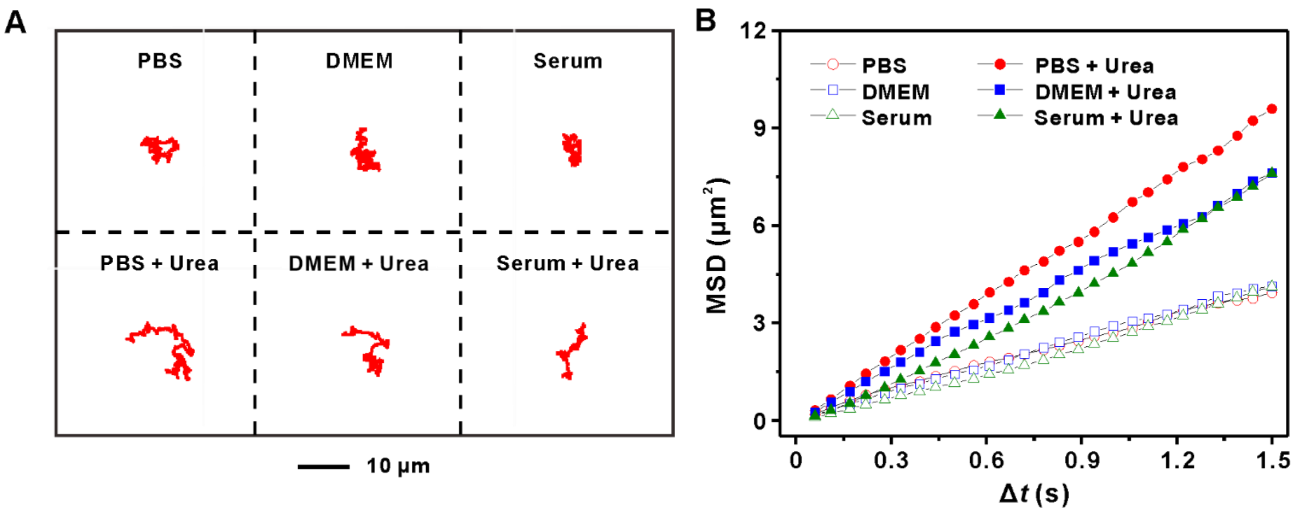

Supplementary Figure 13. Motion behavior of the 50 nm CUPJNRs in different biological media. (A) Typical trajectories of the CUPJNRs at 0 and 20 mM urea concentration over a 10 s period and (B) corresponding MSD of the CUPJNRs *versus* Δ*t*. The trajectories are obtained from Supplementary Video 4.

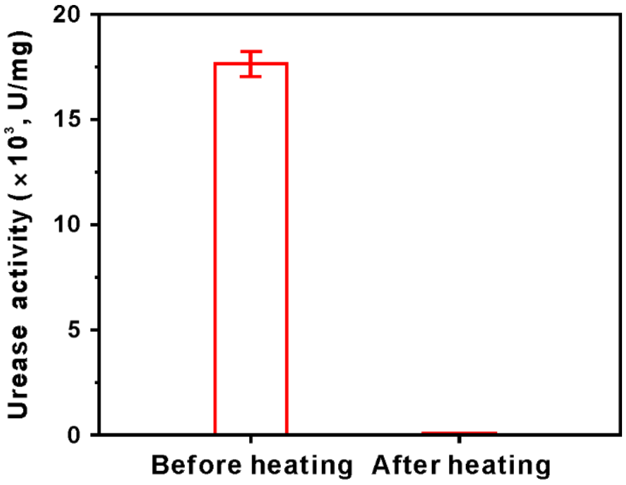

Supplementary Figure 14. The activity of urease (1 μg/mL, dissolved in 10 mM PBS buffer solution, pH 7.4) before and after heating at 95°C for 30 minutes.

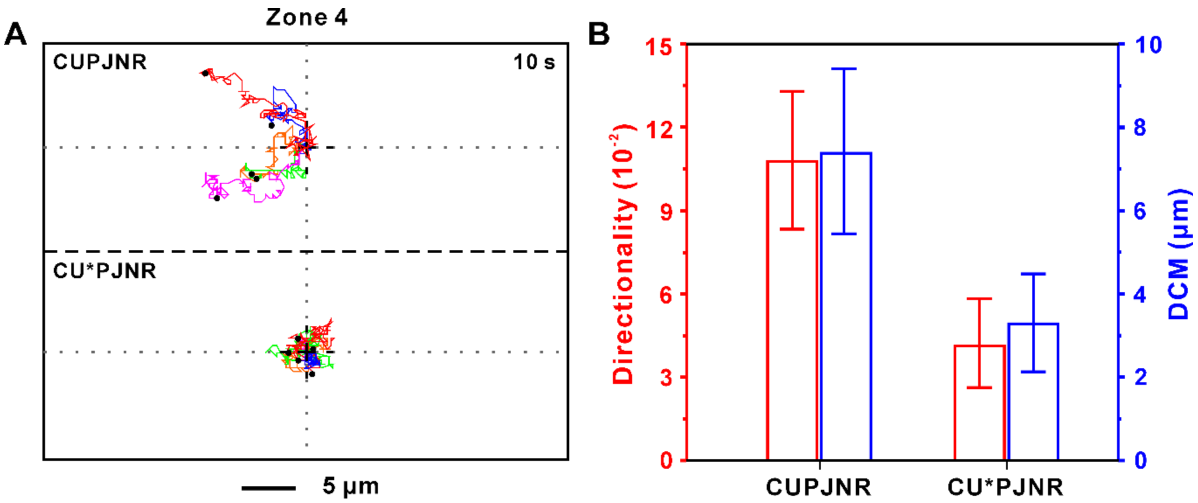

Supplementary Figure 15. Individual chemotactic behavior of the 50 nm CUPJNRs or CU*PJNRs in 3D cell model. (A) Typical trajectories of the CUPJNRs or CU*PJNRs in zone 4 of the microfluidic channel over a 10 s period, and (B) corresponding directionality and DCM [n = 10; mean ± SD]. The trajectories are obtained from Supplementary Video 5.

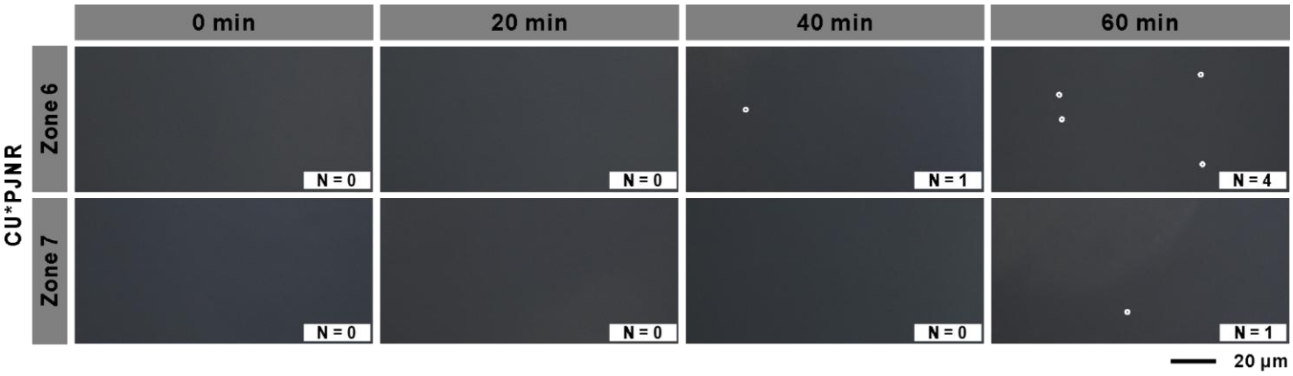

Supplementary Figure 16. Images of zones 6 and 7 of the microfluidic channel at different time points. The white circles represent the CU*PJNRs.

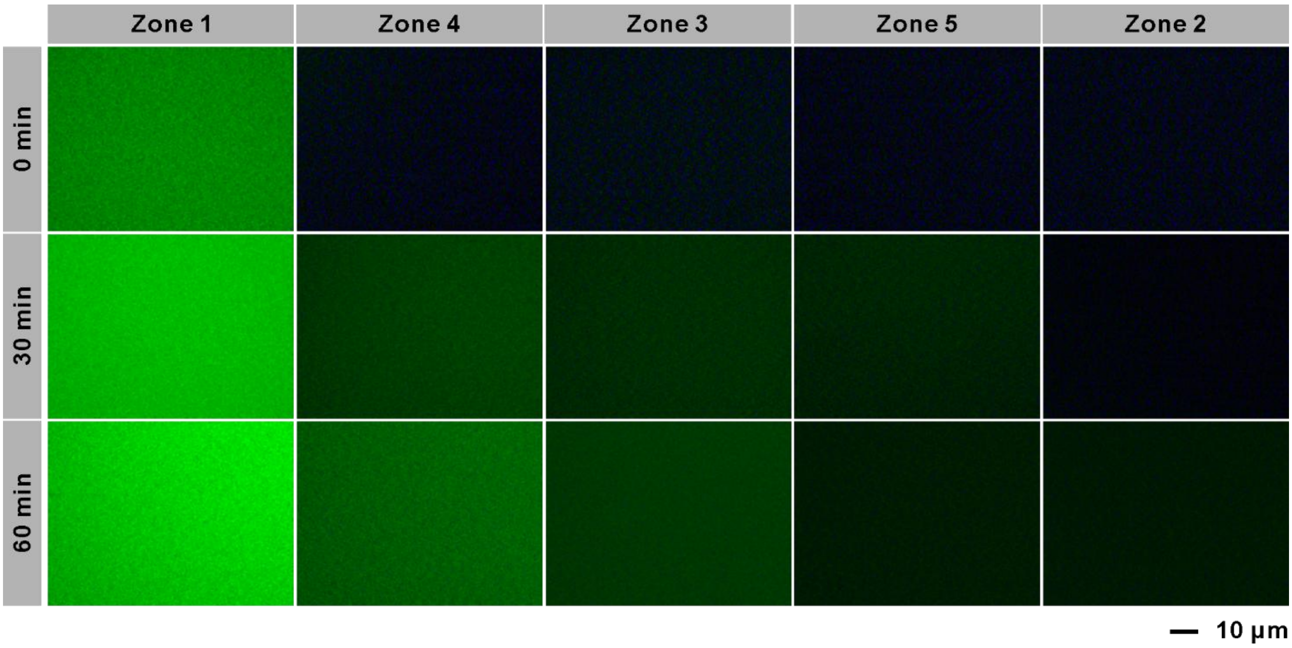

Supplementary Figure 17. Representative fluorescence images of H_2O_2 fluorescent probes at different positions in the microfluidic channel overtime. The left reservoir (zone 1) was filled with collagen gel containing MCF-7 cells, while the right reservoir (zone 2) was filled with collagen gel containing MCF-10A cells. The main channel is 1.8 cm long.

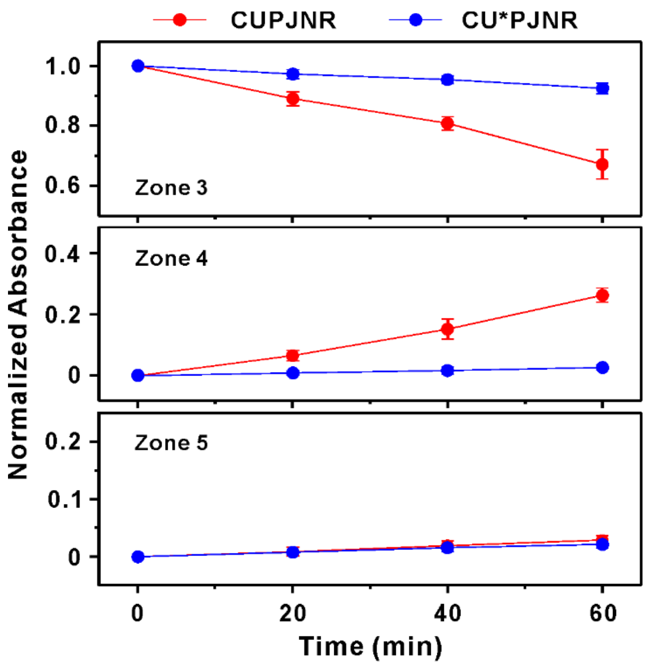

Supplementary Figure 18. Normalized absorbance at 560 nm of the 90 nm CUPJNRs or CU*PJNRs at zones 3–5 of the microfluidic channel within 60 minutes in different situations [n = 3; mean ± SD]. The left reservoir (zone 1) was filled with collagen gel containing MCF-7 cells, while the right reservoir (zone 2) was filled with collagen gel containing MCF-10A cells.

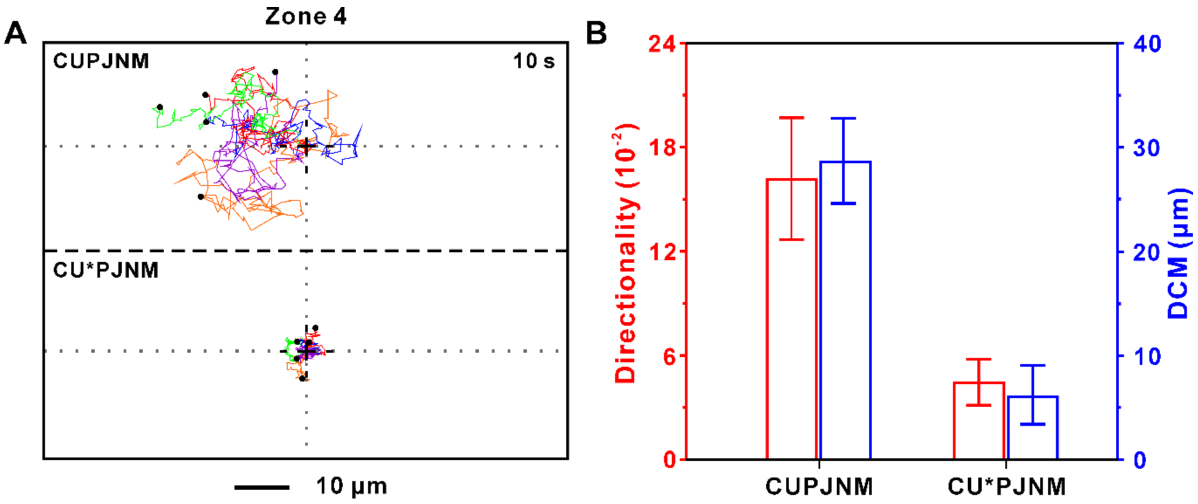

Supplementary Figure 19. Individual chemotactic behavior of the 90 nm CUPJNRs or CU*PJNRs in 3D cell model. (A) Typical trajectories of the CUPJNRs and CU*PJNRs in zone 4 of the microfluidic channel over a 10 s period, and (B) corresponding directionality and DCM [n = 10; mean ± SD]. The left reservoir (zone 1) was filled with collagen gel containing MCF-7 cells, while the right reservoir (zone 2) was filled with collagen gel containing MCF-10A cells. The trajectories are obtained from Supplementary Video 6.

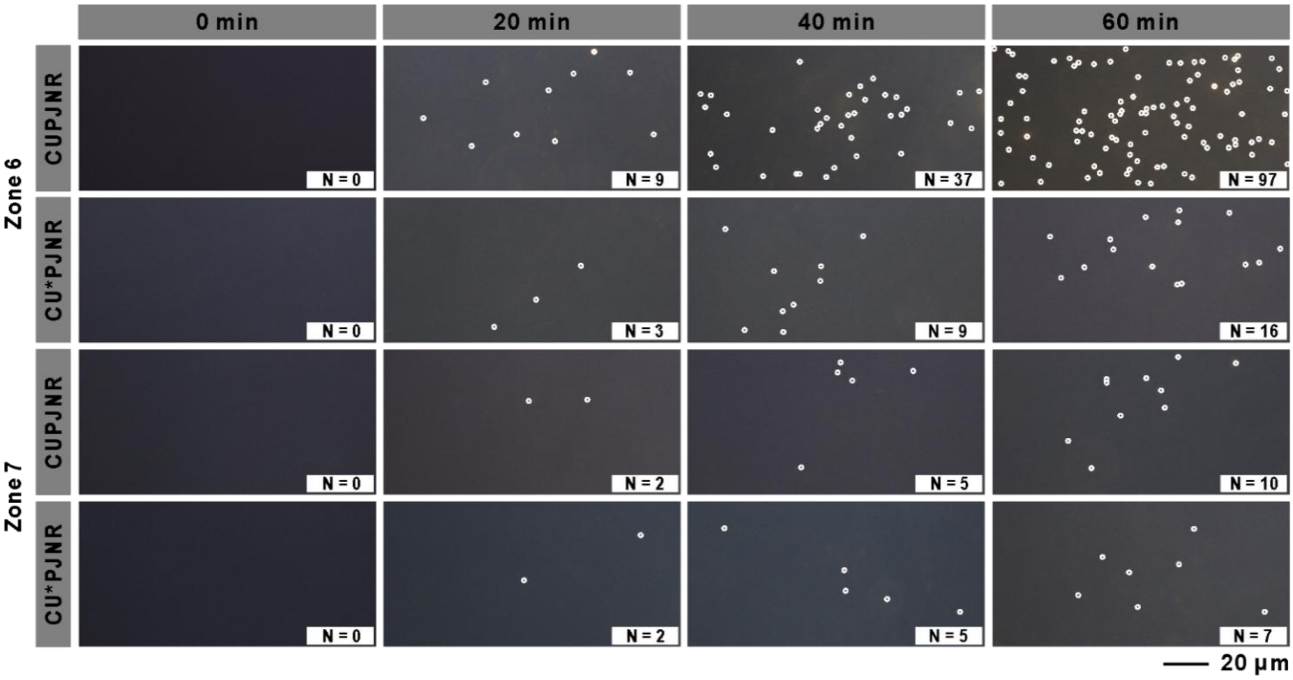

Supplementary Figure 20. Images of zones 6 and 7 of the microfluidic channel at different time points. The left reservoir (zone 1) was filled with collagen gel containing MCF-7 cells, while the right reservoir (zone 2) was filled with collagen gel containing MCF-10A cells. The size of the CUPJNRs or CU*PJNRs is 90 nm. The white circles represent the CU*PJNRs.

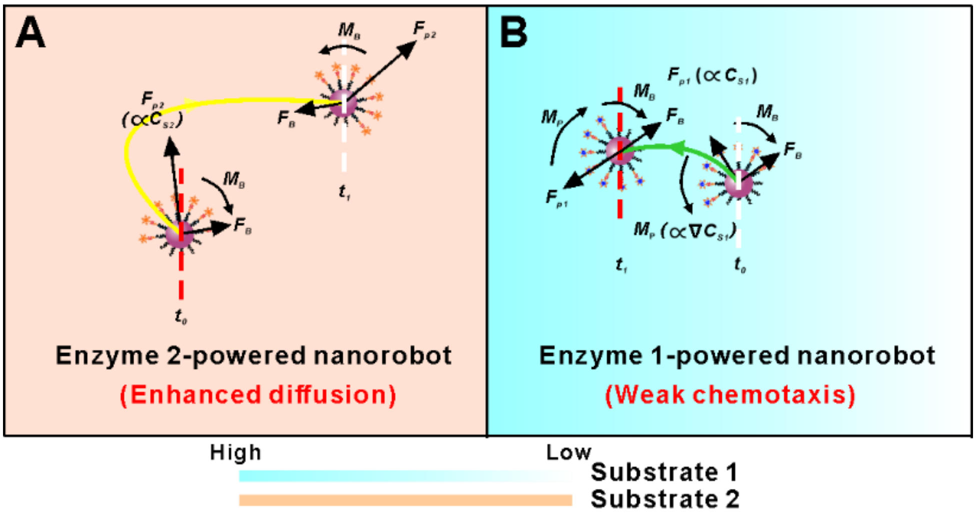

Supplementary Figure 21. Propulsion-enhanced chemotaxis mechanism for (A) single fuel-powered nanorobots, where the fuel shows a concentration high enough to generate strong F_p to counteract $\vec{F}_B(t)$, but with no gradient to generate M_p to counteract $\vec{M}_B(t)$, resulting in enhanced diffusion without directional bias; (B) single enzyme-chemotactic nanorobots, where the fuel concentration gradient is sufficient to induce directional bias, but the fuel concentration is too low to generate translational propulsion, resulting in weak chemotactic behavior.

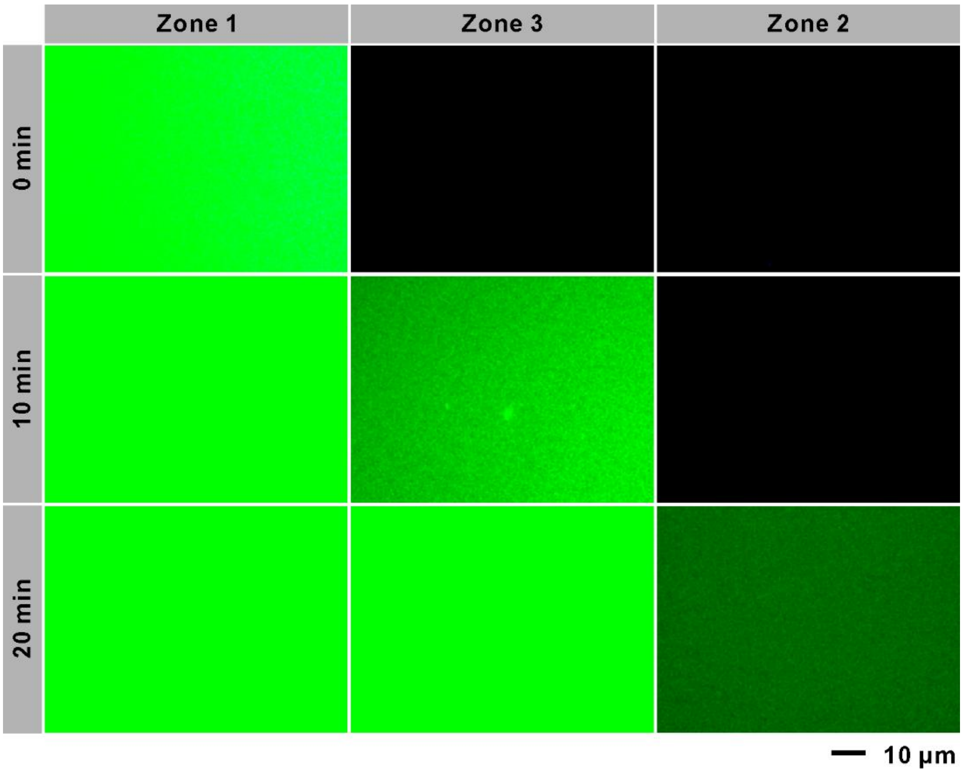

Supplementary Figure 22. Representative fluorescence images for H_2O_2 fluorescent probes at different positions in the *μ*–slide microfluidic channel overtime. The left reservoir (zone 1) was filled with 1 M H_2O_2.

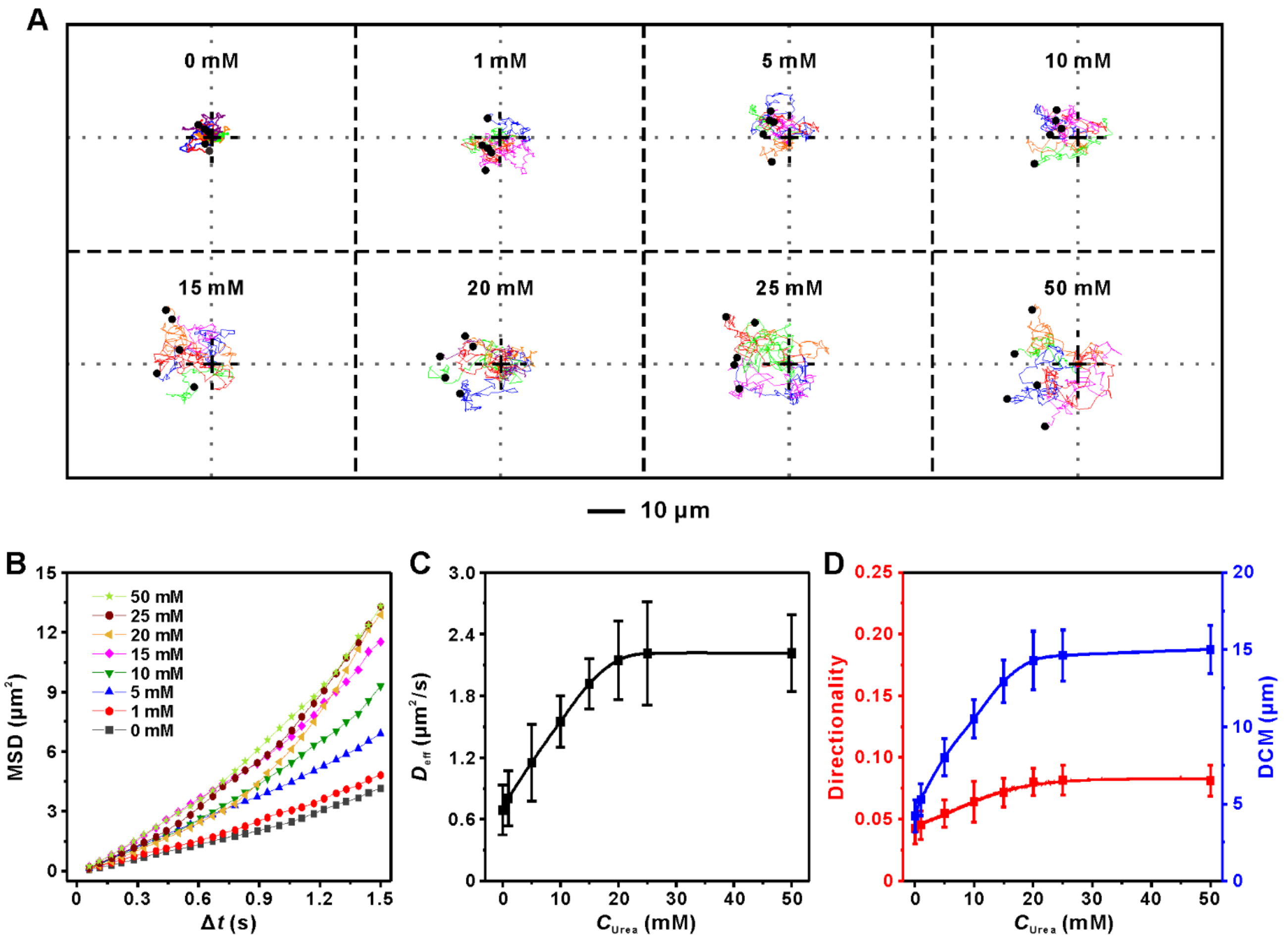

Supplementary Figure 23. Chemotactic behavior of the 50 nm CUPJNRs in a H_2O_2 concentration gradient containing different concentrations of urea. (A) Typical trajectories of the CUPJNRs at different urea concentrations over a 10 s period, (B) corresponding MSD of the CUPJNRs *versus* Δ*t*, (C) corresponding D_{eff} of the CUPJNRs *versus* the urea concentration [n = 10; mean ± SD], and (D) corresponding directionality and displacement of center of mass of the CUPJNRs *versus* the urea concentration [n = 10; mean ± SD]. The trajectories are obtained from Supplementary Video 8.

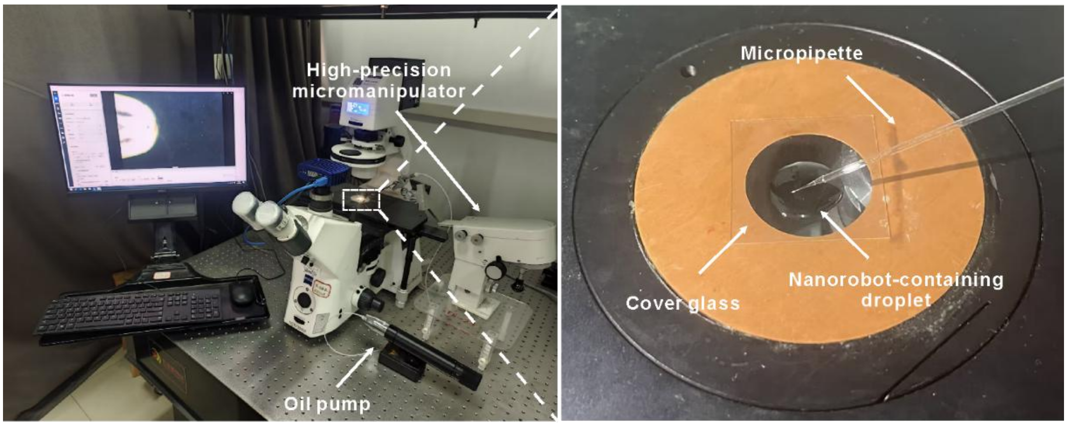

Supplementary Figure 24. Image of the device for evaluating the chemotactic sensitivity of CUPJNRs.

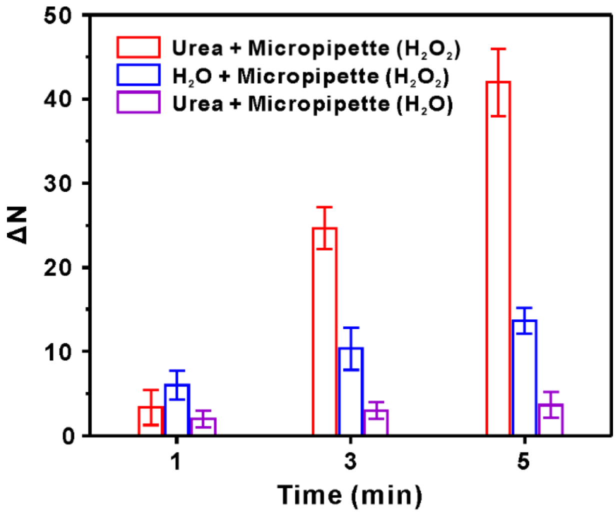

Supplementary Figure 25. The increased number of the 50 nm CUPJNRs in the observation region around the micropipette when the micropipette was filled with 50 μM H_2O_2 within 5 min [n = 3; mean ± SD].

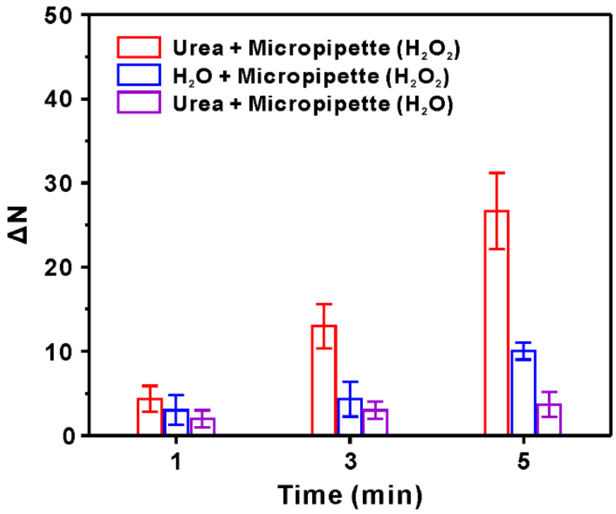

Supplementary Figure 26. The increased number of the 50 nm CUPJNRs in the observation region around the micropipette when the micropipette was filled with 5 μM H_2O_2 within 5 min [n = 3; mean ± SD].

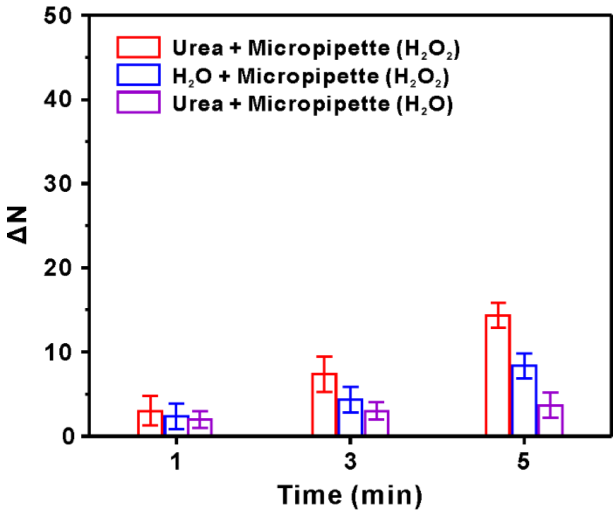

Supplementary Figure 27. The increased number of the 50 nm CUPJNRs in the observation region around the micropipette when the micropipette was filled with 1 μM H_2O_2 within 5 min [n = 3; mean ± SD].

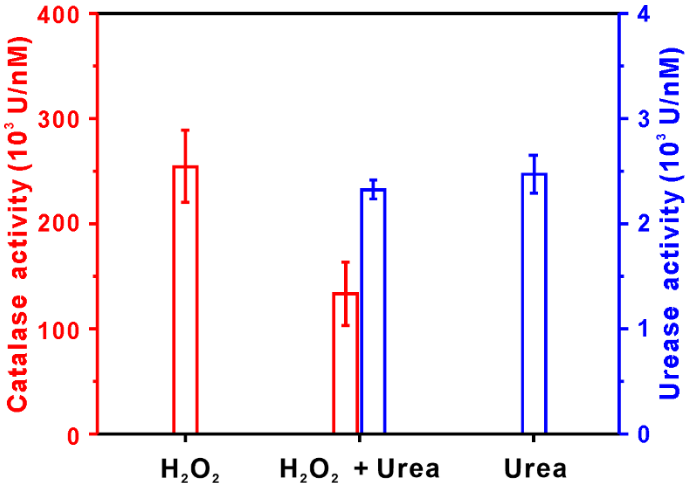

Supplementary Figure 28. The catalytic activity of catalase and urease immobilized on the surface of 50 nm CUPJNR in a single-fuel or mixed-fuel system [n = 3; mean ± SD].

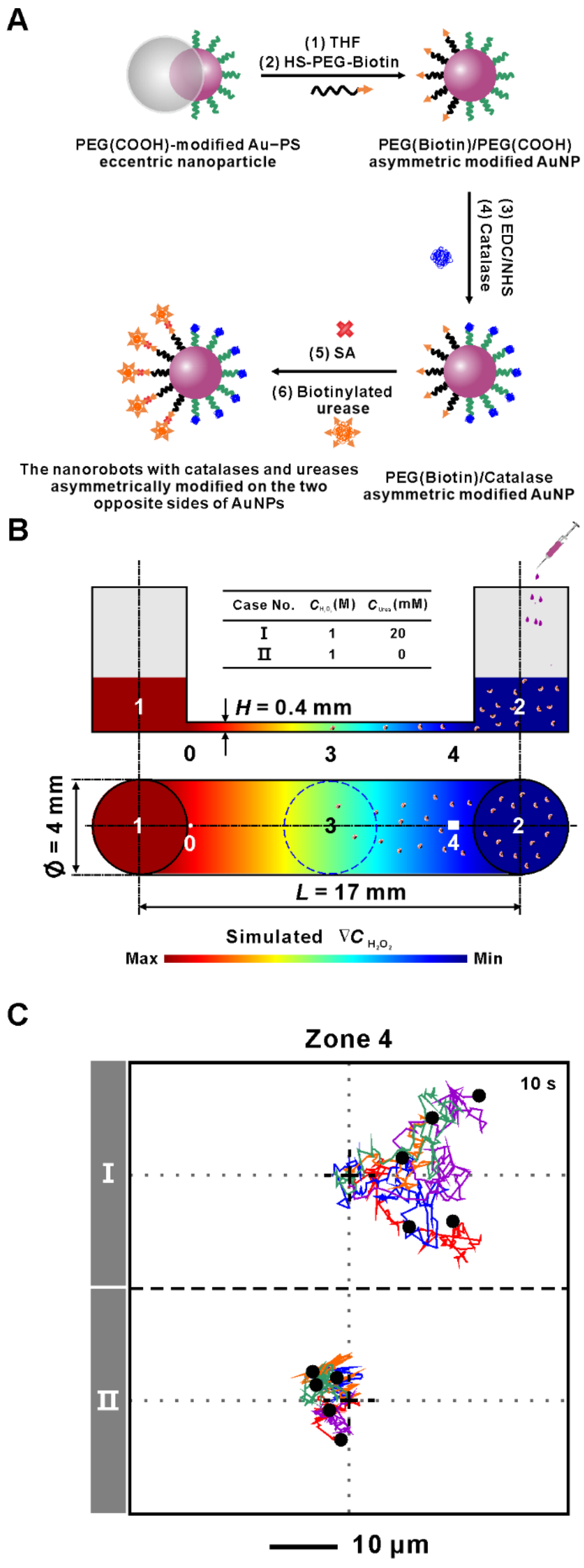

Supplementary Figure 29. Chemotactic behavior of the nanorobots with catalases and ureases asymmetrically modified on the two opposite sides of AuNPs. (A) Schematic illustration of the preparation procedure used to fabricate the nanorobots. (B) Schematic diagram of a μ–slide microfluidic channel with a H_2O_2-containing agarose gel in the left reservoir (zone 1) to establish a H_2O_2 concentration gradient along the straight channel. The nanorobots were added in the right reservoir (zone 2). (C) Typical trajectories of the

nanorobots at zone 4 of the microfluidic channel over a 10 s period in different cases. The trajectories are obtained from Supplementary Video 10.

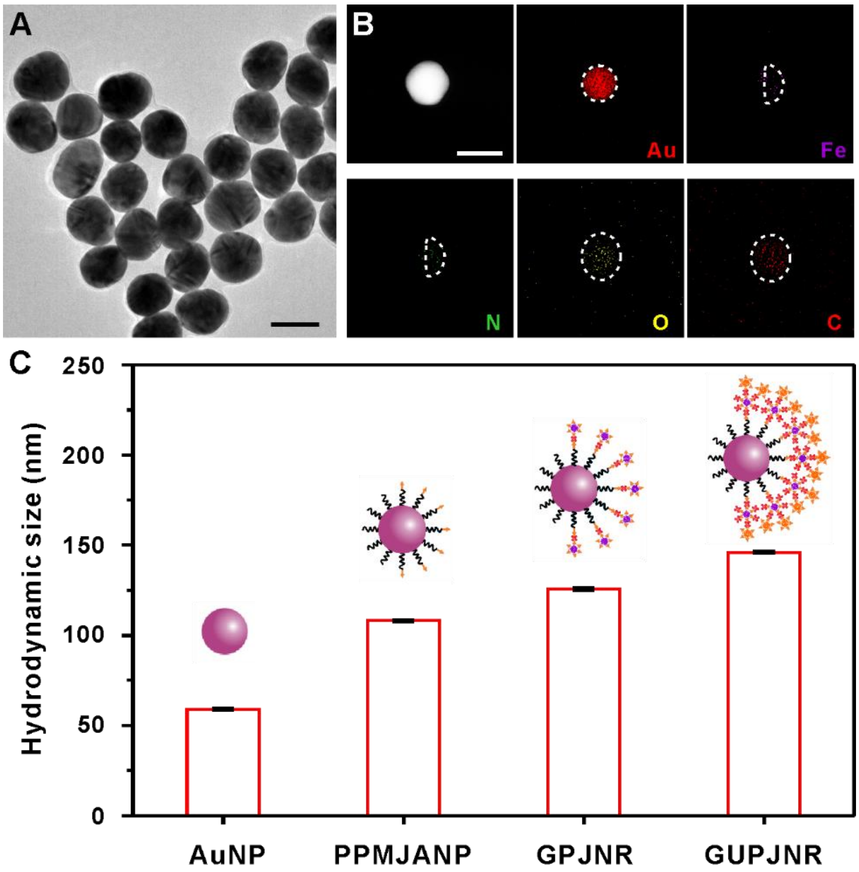

Supplementary Figure 30. Characterization of the glucose oxidase/urease-powered Janus nanorobots (GUPJNRs). (A) TEM image of the GUPJNRs. (B) HAADF-STEM image and energy-dispersive X-ray (EDX) mapping analysis of an individual GUPJNR. Scale bar: 50 nm. (C) Hydrodynamic size of the products during the preparation of the GUPJNRs [n = 3; mean ± SD].

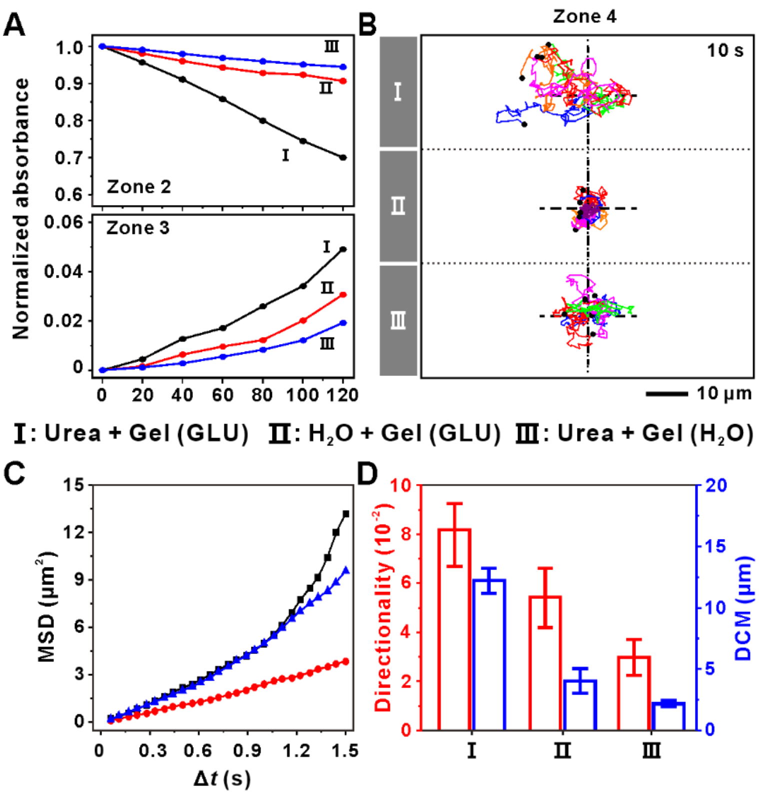

Supplementary Figure 31. Chemotactic behavior of the 50 nm GUPJNRs in a μ–slide microfluidic channel. (A) Normalized absorbance at 530 nm of the GUPJNRs at zones 2 and 3 of the microfluidic channel in Figure 3a within 2 h in different situations. (B) Typical trajectories of the GUPJNRs at zone 4 of the microfluidic channel over a 10 s period in different situations, and (C) corresponding MSD *versus* Δt, and (D) directionality and DCM of the GUPJNRs analyzed from the trajectories. The left reservoir (zone 1) was filled with 1 M glucose. The urea concentration in the solution was 20 mM. The trajectories are obtained from Supplementary Video 11.

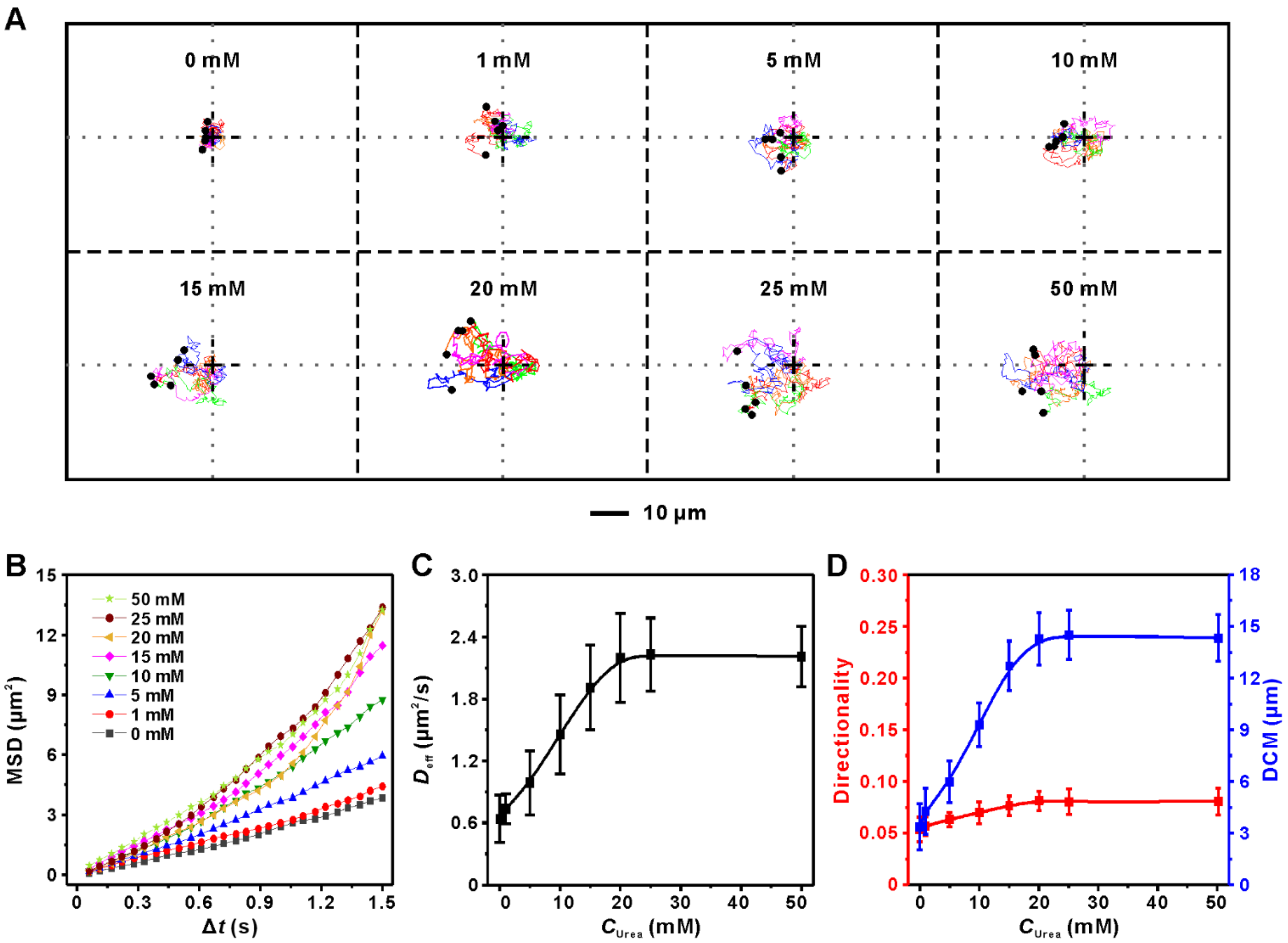

Supplementary Figure 32. Chemotactic behavior of the 50 nm GUPJNRs in a glucose concentration gradient containing different concentrations of urea. (A) Typical trajectories of the GUPJNRs at different urea concentrations over a 10 s period, (B) corresponding MSD of the GUPJNRs *versus* Δt, (C) corresponding D_{eff} of the GUPJNRs *versus* the urea concentration [n = 10; mean ± SD], and (D) corresponding directionality and displacement of center of mass of the GUPJNRs *versus* the urea concentration [n = 10; mean ± SD]. The trajectories are obtained from Supplementary Video 12.

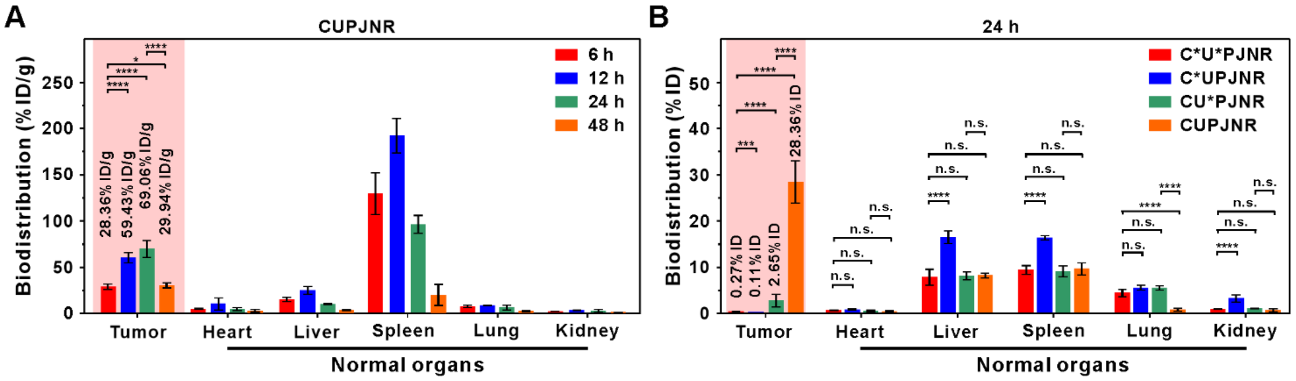

Supplementary Figure 33. *In vivo* targeting accumulation of CUPJNRs. (A) Biodistribution of the CUPJNRs (90 nm) in the tumor-bearing mice model at different time points after intravenous injection of 1.36 billion CUPJNRs [n = 4; mean ± SD]. (B) Biodistribution of the nanorobots with different formulations in the tumor-bearing mice model at 24 h after intravenous injection of 1.36 billion nanorobots [n = 4; mean ± SD]. Statistical comparisons were tested using an ordinary one-way ANOVA with Tukey's multiple-comparisons test. ****P < 0.0001; ***P < 0.001; **P < 0.01; *P < 0.05. The vertical dashed lines indicated the background signals.

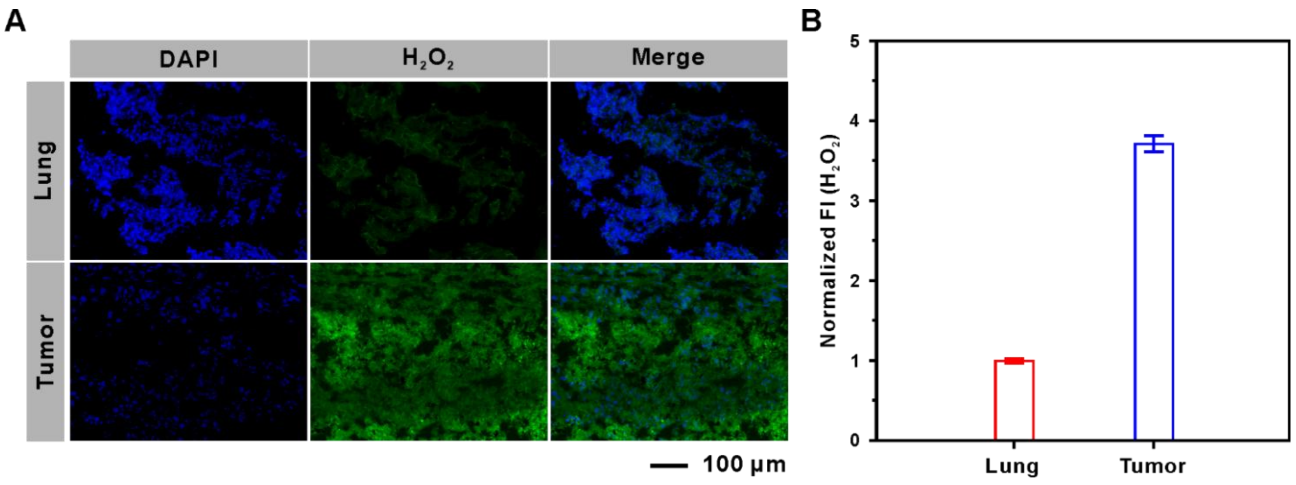

Supplementary Figure 34. (A) Representative H_2O_2 fluorescence staining images of lung and tumor. (B) Normalized of H_2O_2 fluorescence intensity (FI) of lung and tumor [n = 3; mean ± SD].

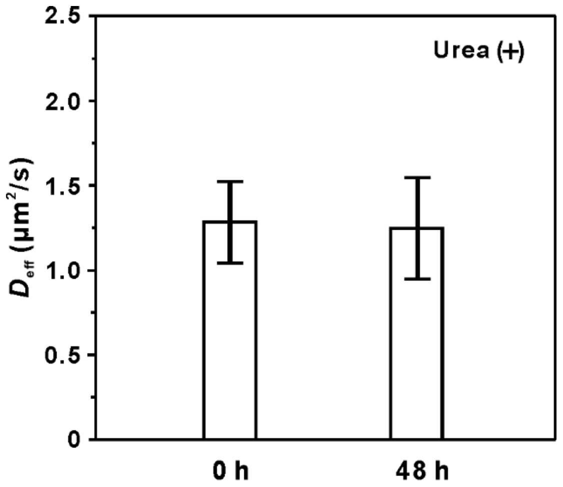

Supplementary Figure 35. The D_{eff} of CUPJNRs after incubation in DMEM for 48 hours [n = 10; mean ± SD]. The concentration of urea was 20 mM.

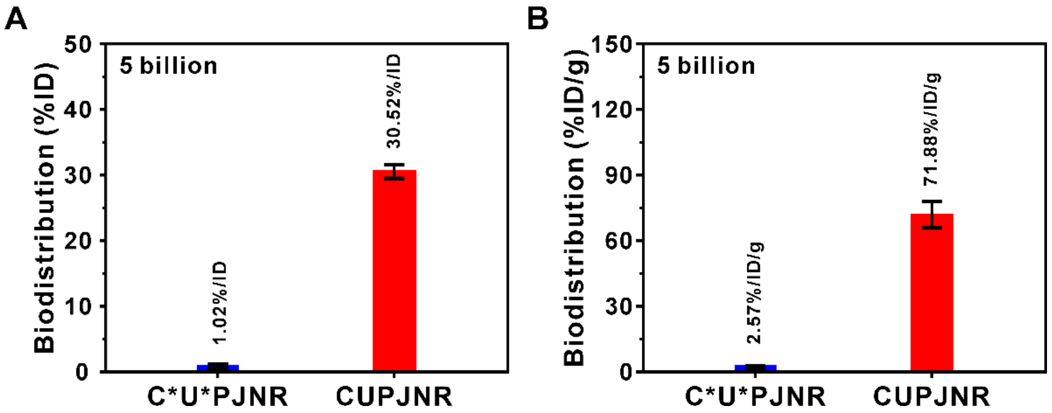

Supplementary Figure 36. Tumor-targeting efficiency of C*U*PJNR and CUPJNR in the tumor-bearing mouse model at 24 hours after intravenous injection of 5 billion nanorobots [n = 4; mean ± SD].

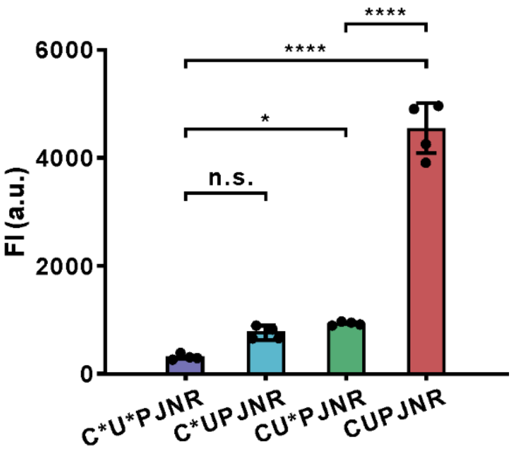

Supplementary Figure 37. The cumulative FI of Cy5 at distances between 0 and 100 μm from the blood vessel [n = 4; mean ± SD].

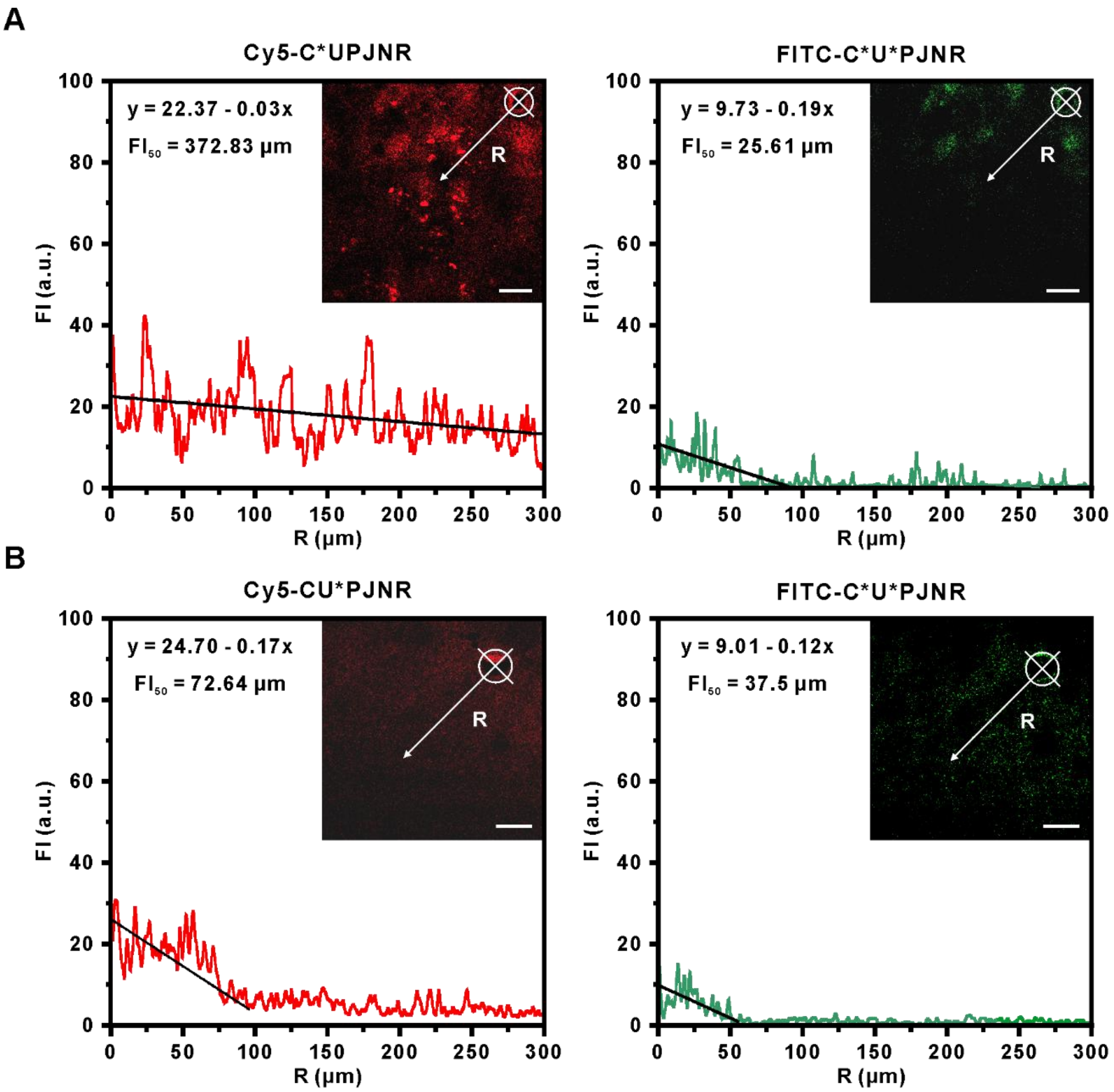

Supplementary Figure 38. The intratumoral penetration capability of nanorobots with different formulations *in vivo*. (A) The red line was the average FI of the Cy5-C*UPJNRs around the injection site, while the black line was the corresponding fitting result. The green line was the average FI of FITC-C*U*PJNRs around the injection site, while the black line was the corresponding fitting result [n = 4]. (B) The red line was the average FI of the Cy5-CU*PJNRs around the injection site, while the black line was the corresponding fitting result. The green line was the average FI of FITC-C*U*PJNRs around the injection site, while the black line was the corresponding fitting result [n = 4]. The point where the FI of nanorobots was dropped to 50% of the injection site was defined as FI_{50}. The insert was the corresponding CLSM image of nanorobots within the tumor tissue. The circle represented the injection site of nanorobots. The FI was analyzed along the direction of white arrow. Scale bar: 50 μm.

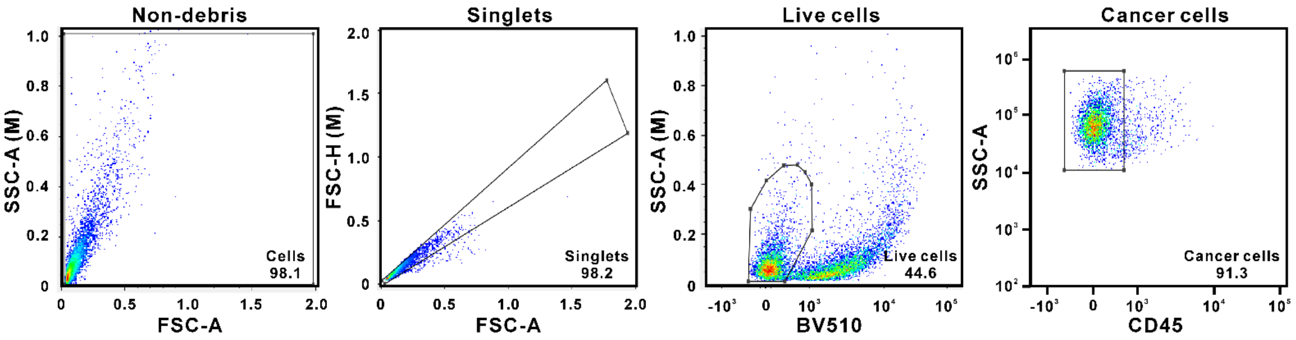

Supplementary Figure 39. Flow cytometry gating strategy for investigating cells that took up nanorobots with different formulations.

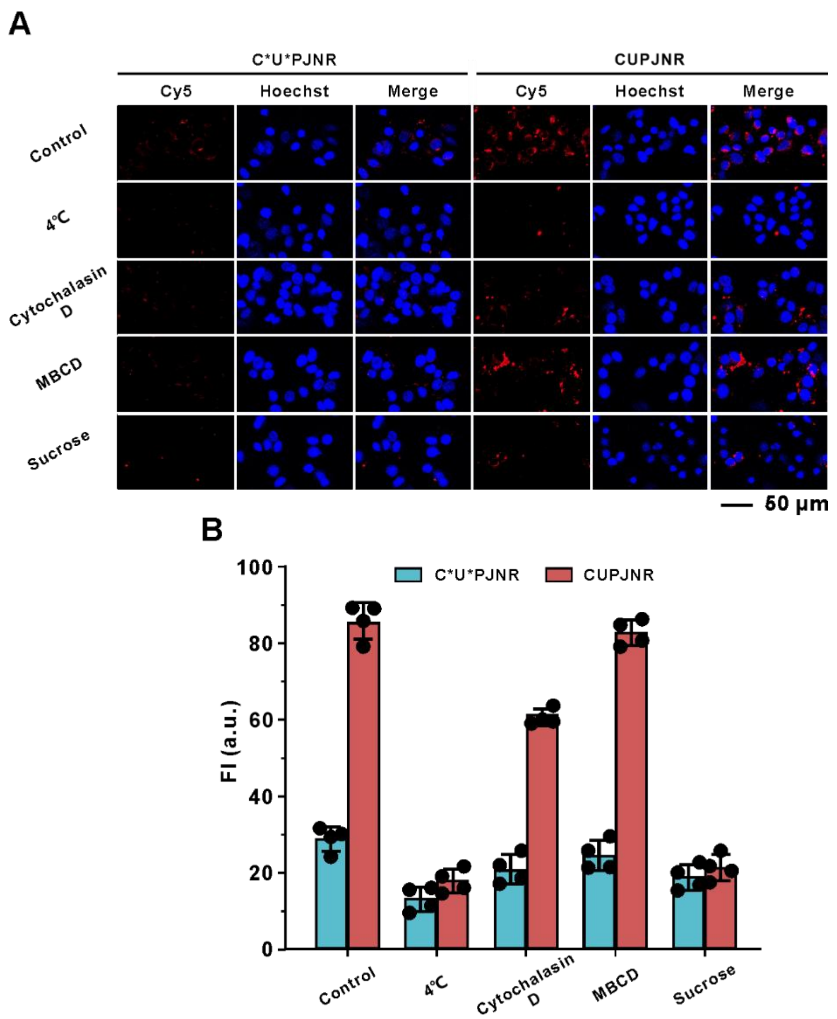

Supplementary Figure 40. Cellular uptake of the C*U*PJNRs and CUPJNRs. (A) Confocal fluorescence images and (B) corresponding FI of Ce6 in MCF-7 cells under indicated conditions [n = 4; mean ± SD]. The urea concentration in DMEM was 10 mM. The C*U*PJNRs and CUPJNRs were pre-modified with Cy5 dyes (red), while the nuclei were stained by Hoechst 33342 (blue). Scale bar: 50 μm. MBCD means methyl-β-cyclodextrin.

A

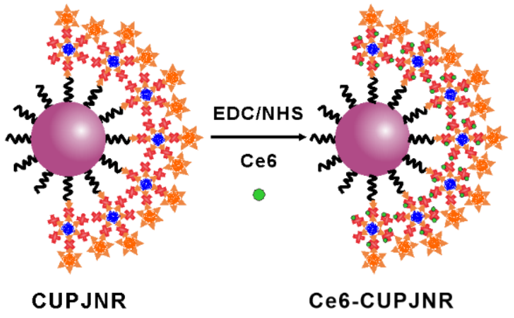

B

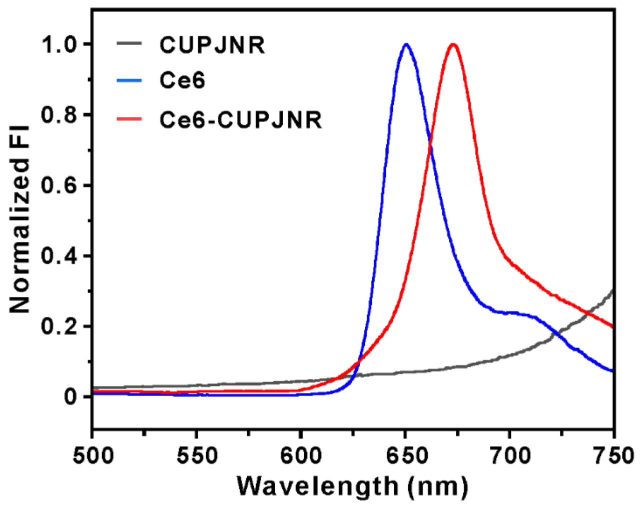

Supplementary Figure 41. Preparation schematic illustration and characterization of Ce6-CUPJNRs. (A) Schematic illustration of the preparation procedure used to modify Ce6 on the surface of CUPJNRs. (B) The normalized fluorescence spectra of CUPJNRs, free Ce6, and Ce6-CUPJNRs.

Discussion:

Ce6 was modified on the surface of nanorobots by using amide bonding between the carboxyl group of Ce6 and the amine groups of SA. After modification of Ce6 on CUPJNRs, a peak of fluorescence emission was observed around 650 nm, suggesting the successful coupling of Ce6 on the surface of CUPJNRs.

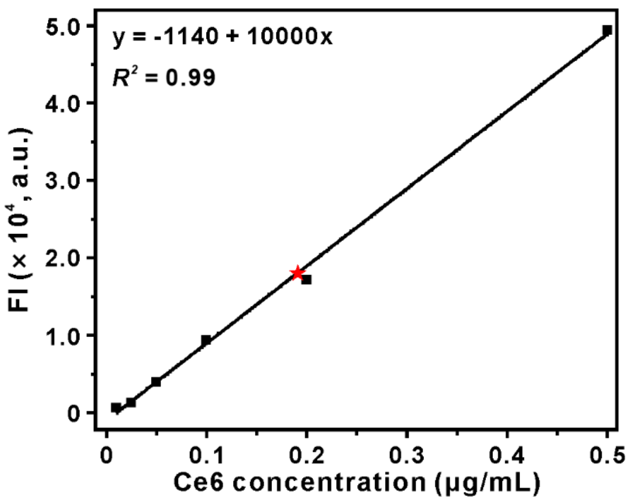

Supplementary Figure 42. The FI of Ce6 at 650 nm *versus* the concentration of Ce6 [n = 4; mean ± SD]. Red asterisk represents the average FI of Ce6 on the surface of 90 nm Ce6-CUPJNR (0.01 nM, 10 μg) [n = 4].

Discussion:

The amount of Ce6 modified on the surface of Ce6-CUPJNR was determined by measuring the fluorescence intensity of 0.01 nM Ce6-CUPJNRs. The result indicates that there was 0.0312 μg of Ce6 modified on the surface of 10 μg of Ce6-CUPJNRs.

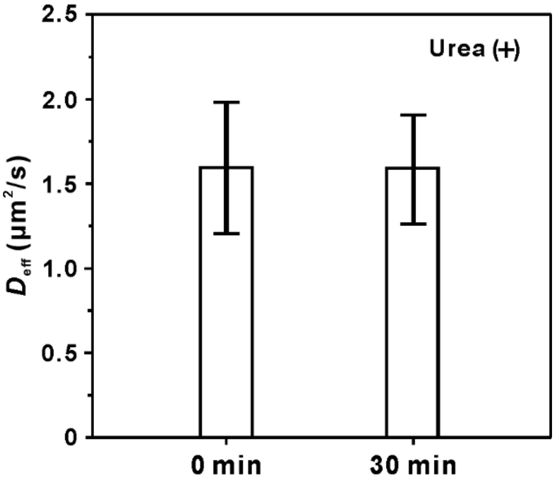

Supplementary Figure 43. The D_{eff} of CUPJNRs after laser irradiation (660 nm, 0.18 W/cm^2) for different time [n = 10; mean ± SD]. The concentration of urea was 20 mM.

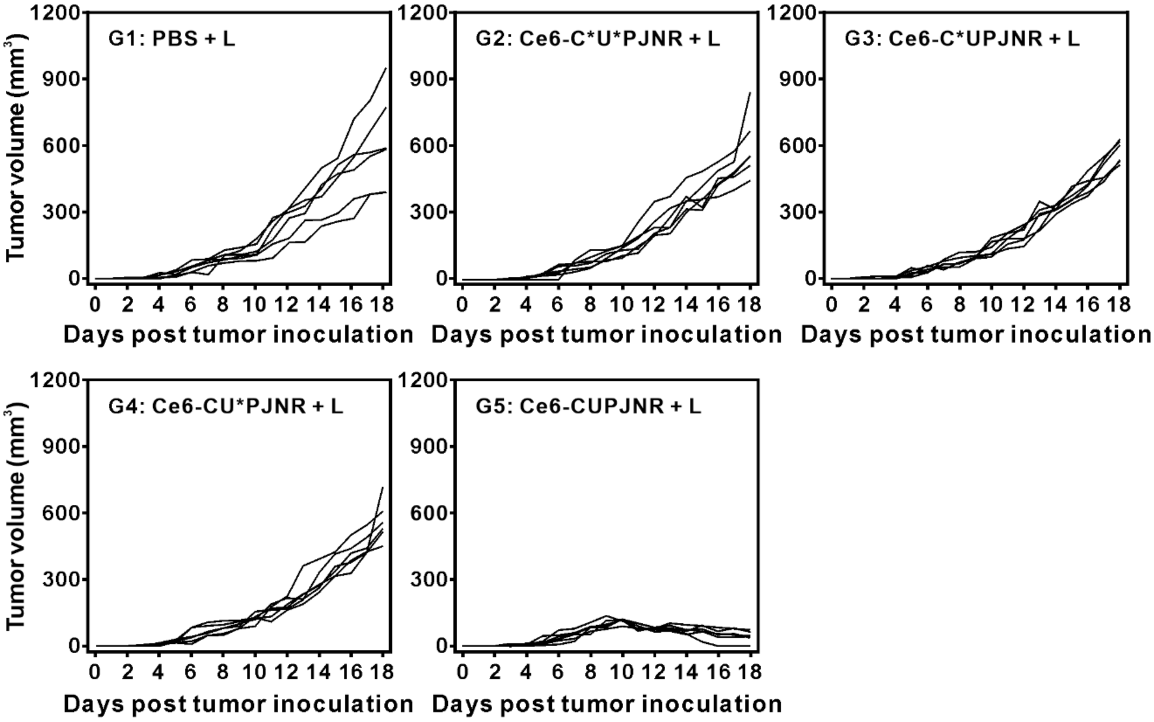

Supplementary Figure 44. Individual tumor growth curves after different treatments.

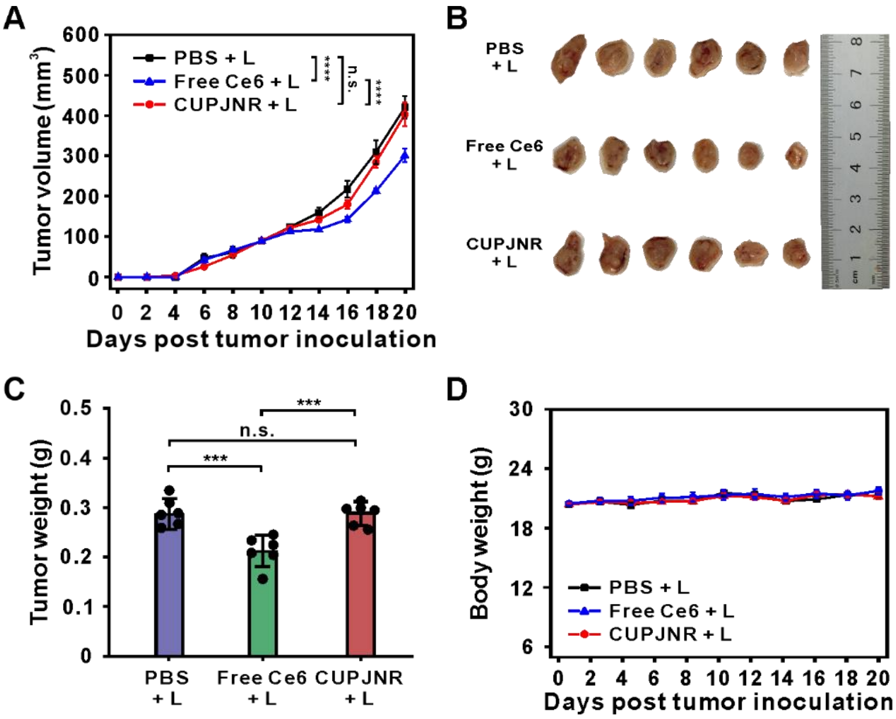

Supplementary Figure 45. Antitumor efficacy of free Ce6 and CUPJNRs *in vivo*. (A) The tumor growth kinetics of tumor-bearing mice that were treated with intravenous injection of PBS, free Ce6 or CUPJNRs every 3 d for a total of 3 doses [n = 6; mean ± SE]. 24 h after injection, the tumor sites were exposed to a 660 nm laser irradiation at a power density of 0.18 W/cm^2 for 10 min. (B) Images of tumors from mice at the end of treatment. (C) Excised tumor weights from mice at the end of treatment [n = 6; mean ± SD]. (D) Body weight of mice with different treatments over the treatment process [n = 6; mean ± SE].

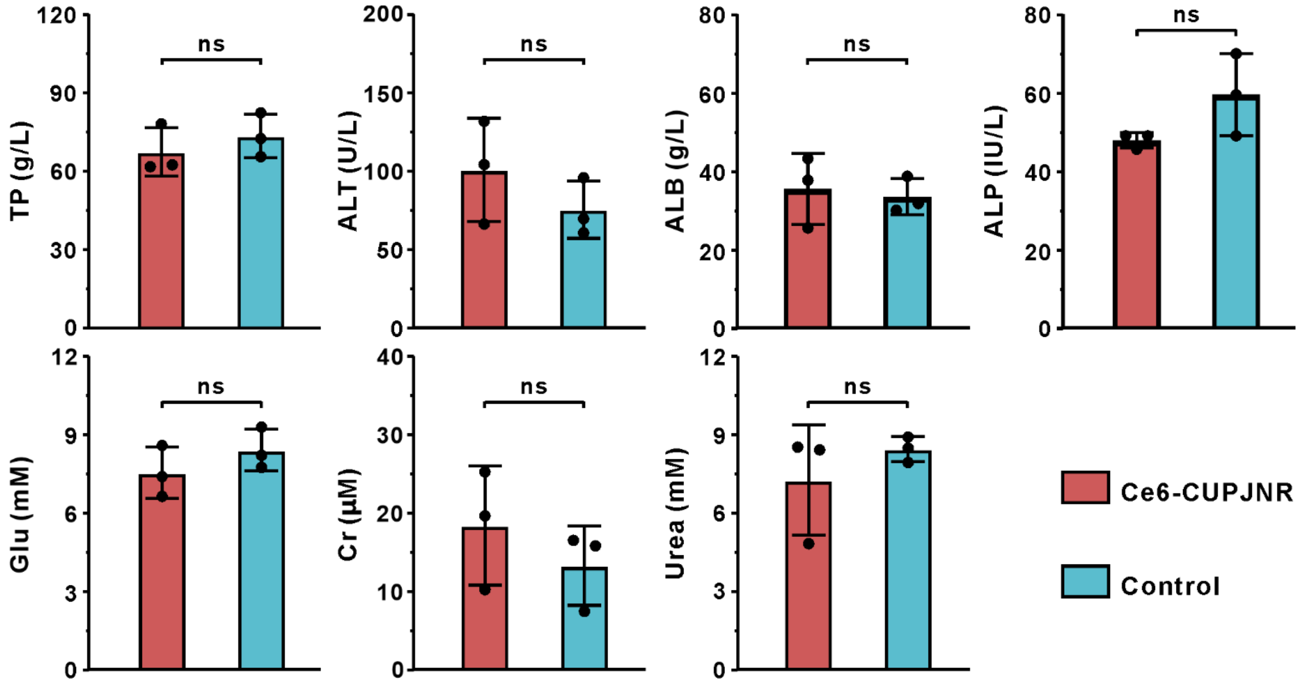

Supplementary Figure 46. Blood chemistry panel of mice non-treated or treated with the Ce6-CUPJNR [n = 3; mean ± SD]. Statistical comparisons were tested using an ordinary one-way ANOVA with Tukey's multiple-comparisons test. n.s., not significant.

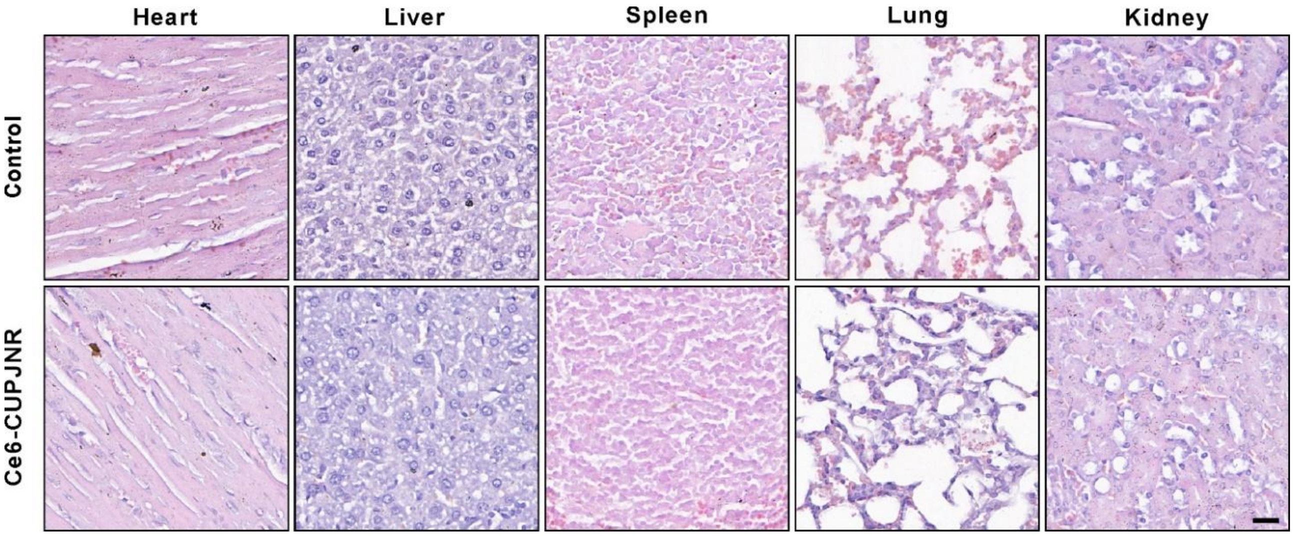

Supplementary Figure 47. Representative photographs of H&E-stained histological sections of major organs from mice non-treated or treated with the Ce6-CUPJNR. Scale bar: 100 μm.

Supplementary Table 1| Summary of the synthetic chemotactic MNMs.

Substrate	Chemoattractant	*In vitro* models		*In vivo* models			Ref.
		Cell types	Targeting efficiency	Disease models	Targeting efficiency	Dose	
$CaCO_3$-based Janus micromotor	pH (6.5–7.4)	Hela cells	×	×	×	×	*Sci. Rep.* **2016**, *6*, 21701
Micromotor coated with FeONPs	NaOH, HCl or HCl and catalase in the H_2O_2 bath	Hela cells	×	×	×	×	*ACS Biomater. Sci. Eng.* **2017**, *3*, 1627−1640
LDH nanosheet-based 2D nanomotor	H_2O_2 (100×10^{-6} M)	MCF-7 cells	×	×	×	×	*Small* **2020**, *16*, e2002732
Supramolecular nanomotor	pH (4.6–7)	Hela cells	×	×	×	×	*Nanoscale* **2020**, *12*, 22495−22501
DNase-functionalized Janus nanomotor	DNA (0.354 μM)	4T1 cells	×	×	×	×	*Nano Lett.* **2021**, *21*, 8086−8094
Nanozyme-powered GNC-Pt nanomotors	H_2O_2 (10%)	HepG2 cells and NCTC1469 cells	×	×	×	×	*Research* **2022**, *21*, 9831012
Enzyme-powered motor	Urea (50 mM)	×	×	Bladder	×	×	*ACS Nano* **2021**, *21*, 11543−11554
Enzyme-powered motor	Glutathione (10 mM)	MCF-7 cells	4.8 times	MCF-7 tumor	×	×	*Sci. China Chem.* **2022**, *65*, 989−1002
ZIF-67@DOX-TPP nanoprobots	H_2O_2 (0.1 mM)	T24 bladder tumor cell	2 times	T24 tumor	×	×	*Sci. Adv.* **2023**, *9*, eadh1736
Pt-powered motor	H_2O_2 (10 mM)	4T1 cells	×	4T1 tumor	×	50 mg/kg	*ACS Appl. Mater. Interfaces* **2022**, *14*, 38172−38184
Enzyme-powered motor	Lactate	×	×	4T1 tumor	~ (1.2 times)	25 mg/kg	*Nano Today* **2022**, *45*, 101542
NO-driven nanomotor	ROS or iNOS	GL261 cancer cell	5.4 times	Brain tumor	~ (3.3 times)	3 mg/mL	*Nat Commun* **2023**, *14*, 941
NO-driven nanomotor	ROS or iNOS	MCF-7 cells	×	Tumor	12.8%ID (7.7 times)	10 mg/kg	*Adv. Mater.* **2022**, *34*, 2206654
Enzyme-powered motor	Inducible nitric oxide synthase or ROS	GL261 cells	×	Glioblastoma	~ (8 times)	~	*Sci. China Chem.* **2024**, *67*, 1277−1288
Bienzyme-powered Janus nanorobots	H_2O_2 (1 μM)	MCF-7 cells	10 times	B16F10 tumor	28.36 %ID	0.5 mg/kg	This work

Supplementary Table 2| Summary of the Ce6-based nanocarrier delivery systems for tumor photodynamic therapy.

Carrier	Injected dose (Ce6)	Power density	Irradiation time	Tumor growth inhibition rate	Ref.
Mesoporous SiO_2 nanoparticles	500–750 μg	0.1 W/cm^2	30 min	~50%	*Cancer Nanotechnol.* **2023**, *14*, 67
Fe_3O_4 nanoparticles	~19.73 μg	0.03 W/cm^2	45 min	~53%	*Nanoscale* **2019**, *11*, 18426−18435
Hyperbranched polyphosphoester nanocarrier	50 μg	0.5 W/cm^2	30 min	~60%	*ACS Appl. Mater. Interfaces* **2018**, *10*, 21198−21205
PEG-Ce6-Gd nanoparticles	200 μg	0.8 W/cm^2	10 min	98%	*Oncol. Rep.* **2021**, *45*, 547−556
Hyaluronic acid (HA)-based micelles	~82 μg	0.05 W/cm^2	24 min	~44%	*Adv. Healthcare Mater.* **2023**, *2302597*
HSA-GOx-CAT nanostructures	~16 μg	0.05 W/cm^2	10 min	~60%	*ACS Appl. Mater. Interfaces* **2022**, *14*, 44029−44038
Pd@Pt nanoplates	~20 μg	0.15 W/cm^2	5 min	~73%	*Adv. Funct. Mater.* **2018**, *28*, 1706310
Bienzyme-powered Janus nanorobots	~0.18 μg	0.18 W/cm^2	30 min	92.7%	This work

References

1. Z. Yang, L. Wang, Z. Gao, X. Hao, M. Luo, Z. Yu, J. Guan, Ultrasmall enzyme-powered Janus nanomotor working in blood circulation system. *ACS Nano* **2023**, 17, 6023−6035.
2. M. Luo, S. Li, J. Wan, C. Yang, B. Chen, J. Guan, Enhanced propulsion of urease-powered micromotors by multilayered assembly of ureases on Janus magnetic microparticles. *Langmuir* **2020**, 36, 7005−7013.
3. M. Alarcón-Correa, J. Günther, J. Troll, V. M. Kadiri, J. Bill, P. Fischer, D. Rothenstein, Self-assembled phage-based colloids for high localized enzymatic activity. *ACS Nano* **2019**, 13, 5810−5815.
4. H. S. Tehrani & A. A. Moosavi-Movahedi, Catalase and its mysteries. *Prog. Biophys. Mol. Bio.* **2018**, 140, 5−12.
5. F. Mou, Q. Xie, J. Feng, S. Che, L. Bahmane, J. Guan, ZnO-based micromotors fueled by CO_2: the first example of self-reorientation-induced biomimetic chemotaxis. *Natl. Sci. Rev.* **2021**, 8, nwab066.
6. E. E. Michaelides, Brownian movement and thermophoresis of nanoparticles in liquids. *Int. J. Heat Mass Tran.* **2015**, 81, 179−187.
